# Functional exploration of copy number alterations in a *Drosophila* model of triple-negative breast cancer

**DOI:** 10.1242/dmm.050191

**Published:** 2024-07-03

**Authors:** Jennifer E. L. Diaz, Vanessa Barcessat, Christian Bahamon, Chana Hecht, Tirtha K. Das, Ross L. Cagan

**Affiliations:** ^1^ Department of Cell, Development, and Regenerative Biology, Icahn School of Medicine at Mount Sinai, New York, NY 10029, USA; ^2^ Internal Medicine, UCLA David Geffen School of Medicine, CA 90095, USA; ^3^ Precision Immunology Institute, Icahn School of Medicine at Mount Sinai, New York, NY 10029, USA; ^4^ School of Cancer Sciences and Wolfson Wohl Cancer Research Centre, University of Glasgow, Glasgow G61 1BD, UK

**Keywords:** *Drosophila*, Triple-negative breast cancer, Genomics

## Abstract

Accounting for 10-20% of breast cancer cases, triple-negative breast cancer (TNBC) is associated with a disproportionate number of breast cancer deaths. One challenge in studying TNBC is its genomic profile: with the exception of TP53 loss, most breast cancer tumors are characterized by a high number of copy number alterations (CNAs), making modeling the disease in whole animals challenging. We computationally analyzed 186 CNA regions previously identified in breast cancer tumors to rank genes within each region by likelihood of acting as a tumor driver. We then used a *Drosophila* p53-Myc TNBC model to identify 48 genes as functional drivers. To demonstrate the utility of this functional database, we established six 3-hit models; altering candidate genes led to increased aspects of transformation as well as resistance to the chemotherapeutic drug fluorouracil. Our work provides a functional database of CNA-associated TNBC drivers, and a template for an integrated computational/whole-animal approach to identify functional drivers of transformation and drug resistance within CNAs in other tumor types.

## Teaser

**Summary:** Modeling of triple-negative breast cancer (TNBC) is challenging owing to the predominance of copy number variations. Here, we combined computational and *Drosophila* modeling to develop a database of functional drivers.

## INTRODUCTION

Breast cancer is the second most common cause of cancer deaths among women in the USA (Siegel et al., 2018). The most aggressive subtype, triple-negative breast cancer (TNBC) makes up ∼15% of breast cancers. TNBC is molecularly heterogeneous with few currently identified druggable molecular targets, poor therapeutic response and low survival rates (Bauer et al., 2007; Dent et al., 2007). Less than 40% of women with metastatic TNBC survive 5 years (Bauer et al., 2007). Standard-of-care treatment of TNBC is limited to chemotherapy, including therapies targeting DNA-damage repair and, for some patients, immunotherapy, including atezolizumab (Marra et al., 2019; Harbeck and Gnant, 2017). Meanwhile, advances in sequencing technology have opened new opportunities for understanding the mechanisms of tumorigenesis and drug response (Hyman et al., 2017; Al-Lazikani et al., 2012). Such studies may improve breast cancer survival by improving predictions of progression in individual patients, identifying novel therapeutic targets and improving the utility of our preclinical models.

Developing genetic models for TNBC is challenging. Most TNBCs contain mutations in *TP53*, but some other genes are also commonly mutated (Cancer Genome Atlas Network, 2012; Jiang et al., 2019; Nagahashi et al., 2018). However, computational work (Cancer Genome Atlas Network, 2012; Jiang et al., 2019; Mermel et al., 2011; Sanchez-Garcia et al., 2014; Curtis et al., 2012) has identified extensive genomic copy number alterations (CNAs) that define regions that commonly overlap between different patients. For example, the *MYC* locus – a key regulator of basal-like tumor biology (Cancer Genome Atlas Network, 2012; Chandriani et al., 2009) – is commonly amplified in TNBC. Understanding the role of CNAs in TNBC would benefit from a comprehensive functional study of putative driver genes and their interactions in a whole animal context.

Recently, the *Drosophila* field has developed multigenic models of cancer to capture aspects of tumor complexity; these models have been used to explore drug responses, including as a screening platform to treat patients with cancer also diagnosed with a resistant disease (Vidal et al., 2005; Markstein et al., 2014; Gladstone et al., 2012; Willoughby et al., 2013; Bangi et al., 2016, 2021, 2019). Although flies lack orthologs of some human tissues, epithelial tissues in *Drosophila*, such as the eye and wing, have proven useful for modeling cancer networks and identifying candidate therapeutics (Vidal et al., 2005; Markstein et al., 2014; Gladstone et al., 2012; Willoughby et al., 2013; Bangi et al., 2016, 2021, 2019; Read et al., 2005; Dar et al., 2012; Hirabayashi et al., 2013; Levinson and Cagan, 2016; Levine and Cagan, 2016).

In this study, we established a *Drosophila* p53-Myc platform as a tool for exploring TNBC genomic complexity. To leverage this platform, we first used a computational approach to rank genes within common TNBC CNA regions based on their likelihood to promote tumor progression. We then used our *Drosophila* p53-Myc platform to assess the functional relevance of many of the most highly ranked candidate genes within each CNA region, based on their ability to enhance transformation in a whole-animal platform. The result is a functional database of TNBC-driving genes within common CNAs. Finally, we used this database to build a library of more-complex *Drosophila* TNBC models. In contrast to a p53-Myc model, these more-complex lines failed to respond to fluorouracil – which is clinically relevant for TNBC – demonstrating that increased genetic complexity can lead to drug resistance and identifying candidate resistance factors. As an integrated approach, this work provides a path towards functionally, deconvoluting the role of CNAs in tumor progression and drug response.

## RESULTS

### *TP53* and *MYC* are the most common driver genes in TNBC

**Fig. 1.**
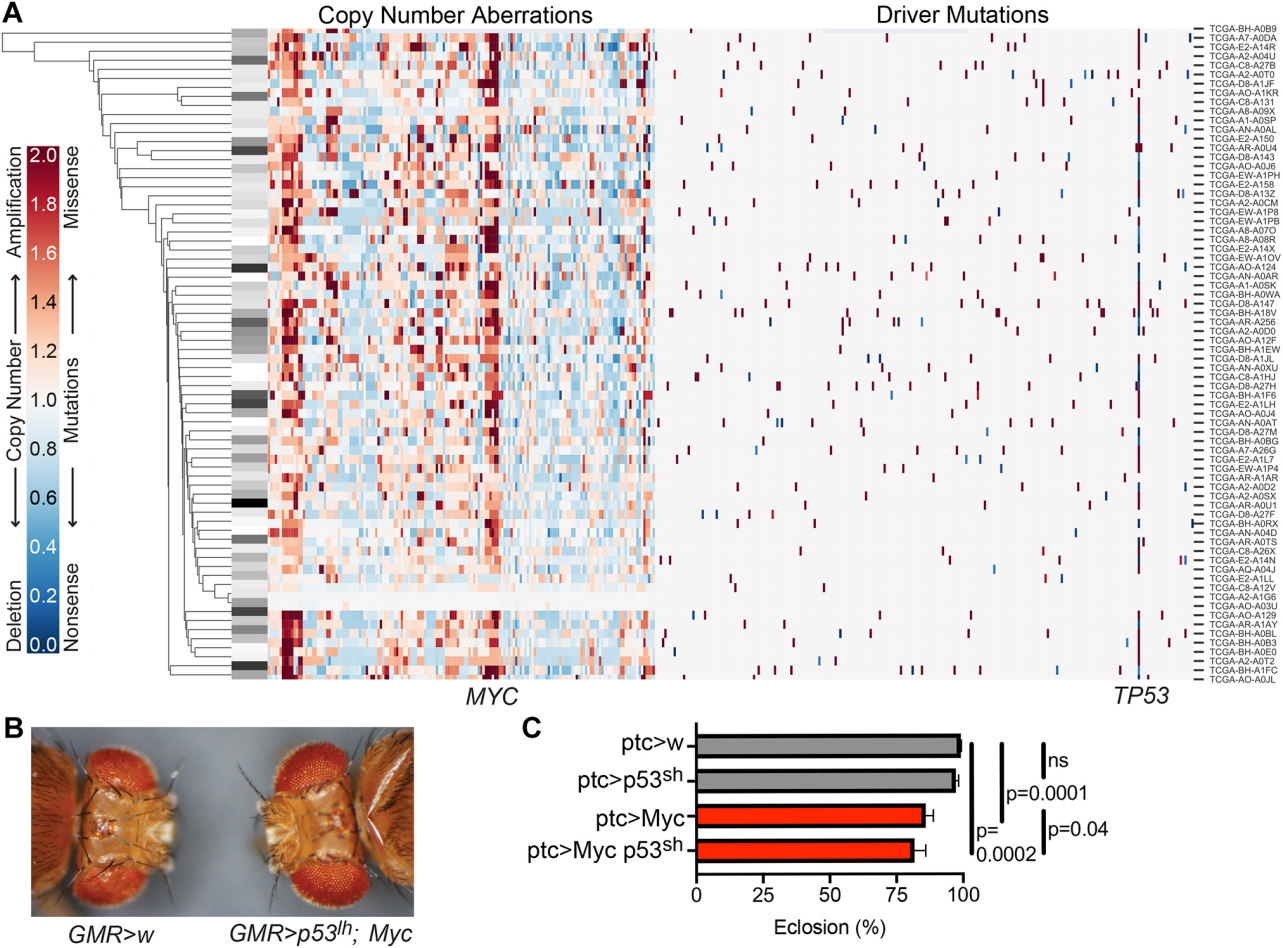
**Altered *TP53* and *MYC* genes are present in TNBC tumors and reduce *Drosophila* survival. (**A) Hierarchical clustering of TNBC primary tumors as listed in TCGA, based on CNA and mutation of putative driver genes. CNAs are shown in order of genomic location, mutated putative driver genes are listed in alphabetical order. Overall survival of each patient is shown coded at the left, with white representing the longest and black the shortest survival. *n=*72. (B) Heads of *GMR>w* (control) and *GMR>p53lh;Myc* flies. Eyes were enlarged when targeted by Myc overexpression plus p53 knockdown (*p53^lh^*; long hairpin). (C) Quantification of survival (shown as percent eclosion) of Myc-expressing flies in the presence (*ptc>w* and *ptc>Myc*) and absence (*ptc>p53^sh^* and *ptc>Myc p53^sh^*) of p53 knockdown (*p53^sh^*; short hairpin). Kruskal–Wallis test: *P*<0.0001. *n=*14. Error bars represent the +standard error of the mean (+s.e.m.) and do not reflect the paired nature of the data. Other *P*-values: Wilcoxon test. See also Fig. S1 and Table S2.

To determine the number of mutated gene loci driving TNBC, we performed an analysis using the MutSigCV algorithm (Lawrence et al., 2013; https://www.genepattern.org/modules/docs/MutSigCV#gsc.tab=0). We found that 81% of TNBC tumors in the 2012 The Cancer Genome Atlas (TCGA) dataset (Cancer Genome Atlas Network, 2012) include a mutation predicted to alter *TP53* function, which is consistent with the reported 80% of basal-like tumors (Cancer Genome Atlas Network, 2012). No other driver genes were commonly mutated (Fig. 1A; Table S2). This is consistent with previous work showing that breast cancer is primarily driven by CNAs rather than point mutations (Jiang et al., 2019; Ciriello et al., 2013).

The location and size of a CNA is not influenced solely by selective pressure, but also by chromatin architecture and the location of recombination hotspots (Pfeifer et al., 2006; Taylor et al., 2008; Li et al., 2014). As a result, a small number of genes within a given CNA region are responsible for the selective pressure and are therefore ‘drivers’, while neighboring genes are included due to proximity to the driver and mostly represent ‘passengers’ that do not appreciably contribute to disease progression.

As a first step towards exploring the genes within CNAs from the TCGA data, we began with 186 CNAs previously identified by GISTIC 2.0 and ISAR (Cancer Genome Atlas Network, 2012; Sanchez-Garcia et al., 2014, Mermel et al, 2011). We performed hierarchical clustering using Euclidean distance on the 186 CNAs and driver gene mutations for the 72 primary TNBC tumors with both types of data available. We found that the tumors did not fall into discrete clusters, and tumors from patients with shorter or longer overall survival did not cluster together (Fig. 1A). This suggests that in the aggregate, these genomic alterations do not explain differences in clinical outcome. This may be because some CNAs have more impact on outcomes than others or because region-level analysis does not provide the resolution to understand the impact of individual driver genes. Therefore, a key step in using this genomic data to model TNBC is to identify drivers within the common CNAs.

The most common TNBC CNA was amplification of 8q24.1, a small region that – as identified by GISTIC 2.0 – solely contains the oncogene *MYC* (Fig. 1A). Excluding combinations of CNA that overlap, the most frequently identified combination of two events was mutation of *TP53* and amplification of *MYC*, occurring in 72% of TNBC tumors. We, therefore, used both *TP53* and *MYC* as the basis of a *Drosophila* platform designed to identify additional drivers.

### p53 and Myc promote cancer-like phenotypes in *Drosophila*

To characterize phenotypes that are due to mutations in p53 and Myc, we generated individual fly lines containing transgenes that provide targeted expression of (i) *Drosophila* Myc (*UAS-Myc*) plus (ii) two different RNA-interference-mediated knockdown constructs targeting endogenous P53 (*UAS-p53^lh^* or *UAS-p53^sh^*) (see Fly stocks section in Materials and Methods for details). Overexpression of Myc and strong loss of P53 (∼80% in the presence of *UAS-Myc*) was confirmed by western blotting (Figs S1A, B). To confirm activity, we used a *GMR-GAL4* driver to express *UAS-p53^lh^* plus *UAS-Myc* (yielding *GMR>p53^lh^;Myc* flies) in the developing eye field. *GMR>p53^lh^;Myc* flies exhibited enlarged eyes with normal ommatidial patterning (Fig. 1B), presumably reflecting Myc-mediated cell enlargement.

We also tested the p53^sh^ and Myc transgenes individually and in combination with a *patched-Gal4* driver (*ptc*) that directs discreet expression of upstream activating sequence (*UAS*)-fused transgenes during several developmental stages. *ptc>Myc* pupal survival (the percentage of pupae that survive to adulthood, also known as the eclosion rate; see Lethality analyses in Materials and Methods) was reduced to 85.8%. Further reduced survival (81.5%) was exhibited by *ptc>Myc,p53^sh^* (Fig. 1C). Reducing P53 alone (*ptc>p53^sh^*) had no effect on survival. Similar results were obtained in the background of another fly strain (*yhsf*) (see Fly stocks in Materials and Methods), and with *p53^lh^* (Fig. S1C,D).

**Fig. 2.**
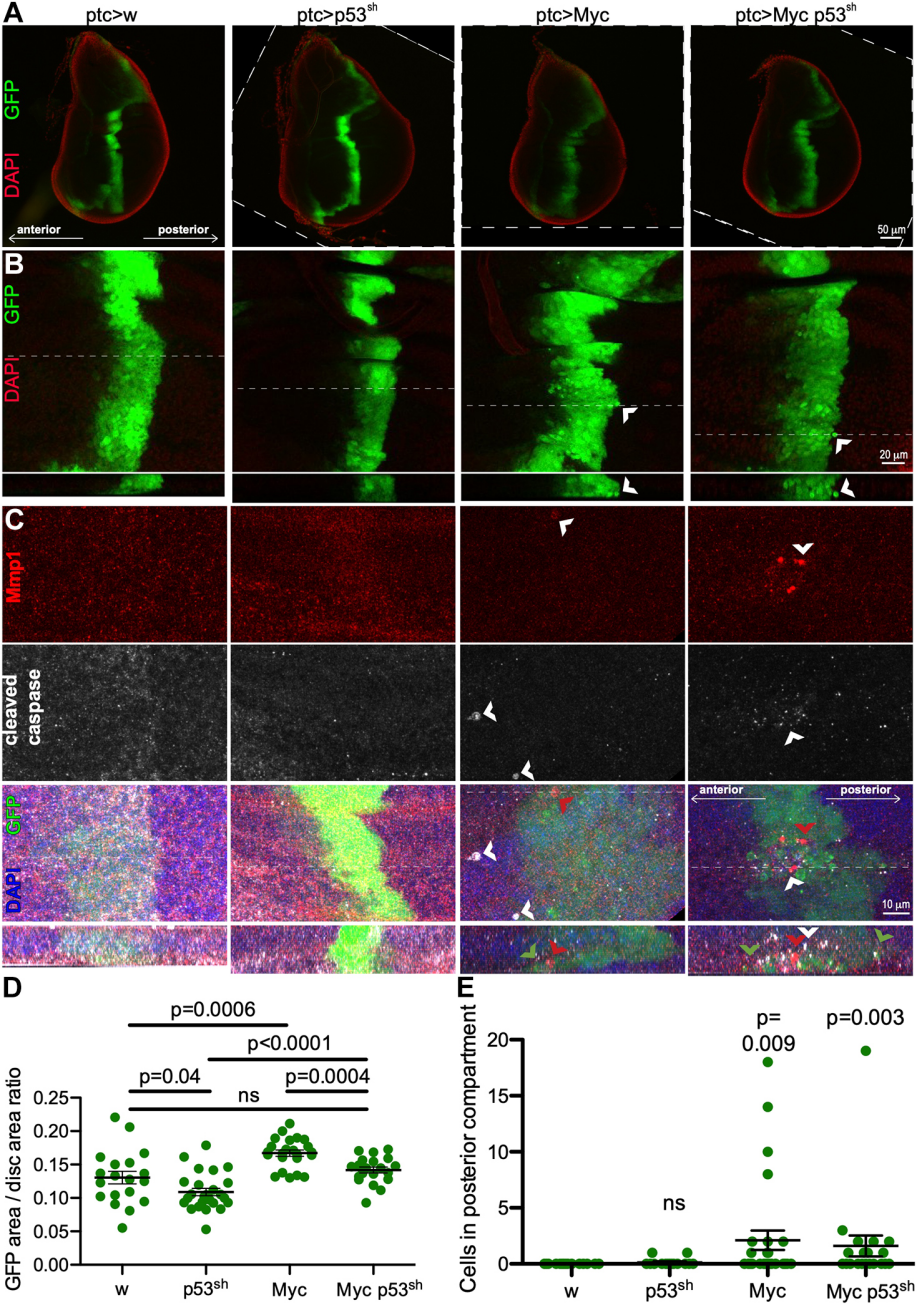
**Overexpression of Myc promotes tissue expansion and cell translocation in *Drosophila* wing discs.** (A) Representative wing discs of flies expressing combinations of *Myc* and *p53^sh^* as indicated. Genotypically *white* (*ptc>w*) flies served as controls. DAPI staining (red) highlights tissue boundary, GFP signal (green) demarcates transgene expression. Some images rotated for comparison with borders indicated by dashed lines. (B) Maximum projections of confocal *z*-stacks of the lower half of the wing discs as in shown in the respective images in A, dashed lines indicate the region of virtual sectioning shown in lower inset. (C) Maximum projections (upper three rows) and *z*-stacks (bottom row) of confocal stacks of the lower half of wing discs such as in A stained with antibodies against Mmp1 antibody (red) or cleaved-caspase (white), both indicative of cell translocation (Rudrapatna et al., 2013); dashed lines indicate the region of virtual sectioning shown in lower inset. Arrowheads in B and C mark delaminating cells. Brightness and contrast were uniformly increased to improve visualization. In A-C, anterior at left, posterior at right, apical at top, basal at bottom. (D) Quantification of transgenic tissue overgrowth in flies expressing *Myc* and *p53^sh^* driven by *ptc-Gal4* alone or in combination. Kruskal–Wallis test: *P*<0.0001. Other *P*-values: Student's *t*-tests. (E) Quantification of cell translocation in transgenic tissue produced by flies expressing *Myc* and *p53^sh^* driven by *ptc-Gal4* or in combination. Kruskal–Wallis test: *P*=0.0075. Other *P*-values: Mann–Whitney test compared to *w* controls. No significant differences were observed between flies expressing *Myc* and *Myc,p53^sh^*. See also Fig. S2.

Using an inducible *UAS-GFP* to visualize transformed cells, we observed expansion of the *ptc* domain at the anterior/posterior boundary of developing *ptc>Myc* wing epithelia (‘wing discs’; Fig. 2A,D, Fig. S2B). In contrast, the *ptc* domain was smaller with knockdown of p53 by *p53^sh^* (Fig. 2A,D) and *p53^lh^* (Fig. S2B). In the presence of *ptc>Myc*, *p53^sh^* restored the *ptc* domain area to normal size (*ptc>Myc,p53^sh^*; Fig. 2D). We have previously observed that p53 knockdown can reduce transformation phenotypes in the presence of an oncogene, although it enhances overall transformation by reducing senescence (Bangi et al., 2016). Area reduction by *p53^lh^* was not significant (Fig. S2B).

Specific targeting of transgene expression to the anterior/posterior boundary of the wing disc provides a useful assay for measuring aspects of transformation in *Drosophila* cancer models (Levinson and Cagan, 2016; Vidal et al., 2006; Sonoshita et al., 2018). In confocal images of both *ptc>Myc* and *ptc>Myc,p53^sh^* wing epithelia, we observed transformed cells delaminating into the basal region of the epithelium (Fig. 2B). These delaminating cells showed high levels of cleaved, activated caspase and matrix metalloproteinase (Fig. 2C). Some of the delaminating cells were seen migrating away from the *ptc* domain (Fig. 2E). Delaminating, caspase-positive cells (Fig. S2A) and migrating cells (Fig. S2C) were also seen with *ptc>p53^lh^;Myc*. The co-occurrence of caspase activation with translocation of cells away from the *ptc* domain is consistent with studies in other *Drosophila* cancer models (Geisbrecht and Montell, 2004; Rudrapatna et al., 2013; Gorelick-Ashkenazi et al., 2018), in which this translocation was preceded by aspects of transformation and epithelial-to-mesenchymal transition. Altering additional cancer genes enhanced this phenotype (below), further suggesting these are, indeed, aspects of transformation. However, more-detailed studies will be required to confirm this. Based on the multiple aspects of transformation exhibited by our *ptc>Myc,p53^sh^* line, we concluded that this *Drosophila* line provides a useful genetic platform for identifying functional candidate driver genes within regions of CNA.

### Prioritizing candidate driver genes from CNAs

**Fig. 3.**
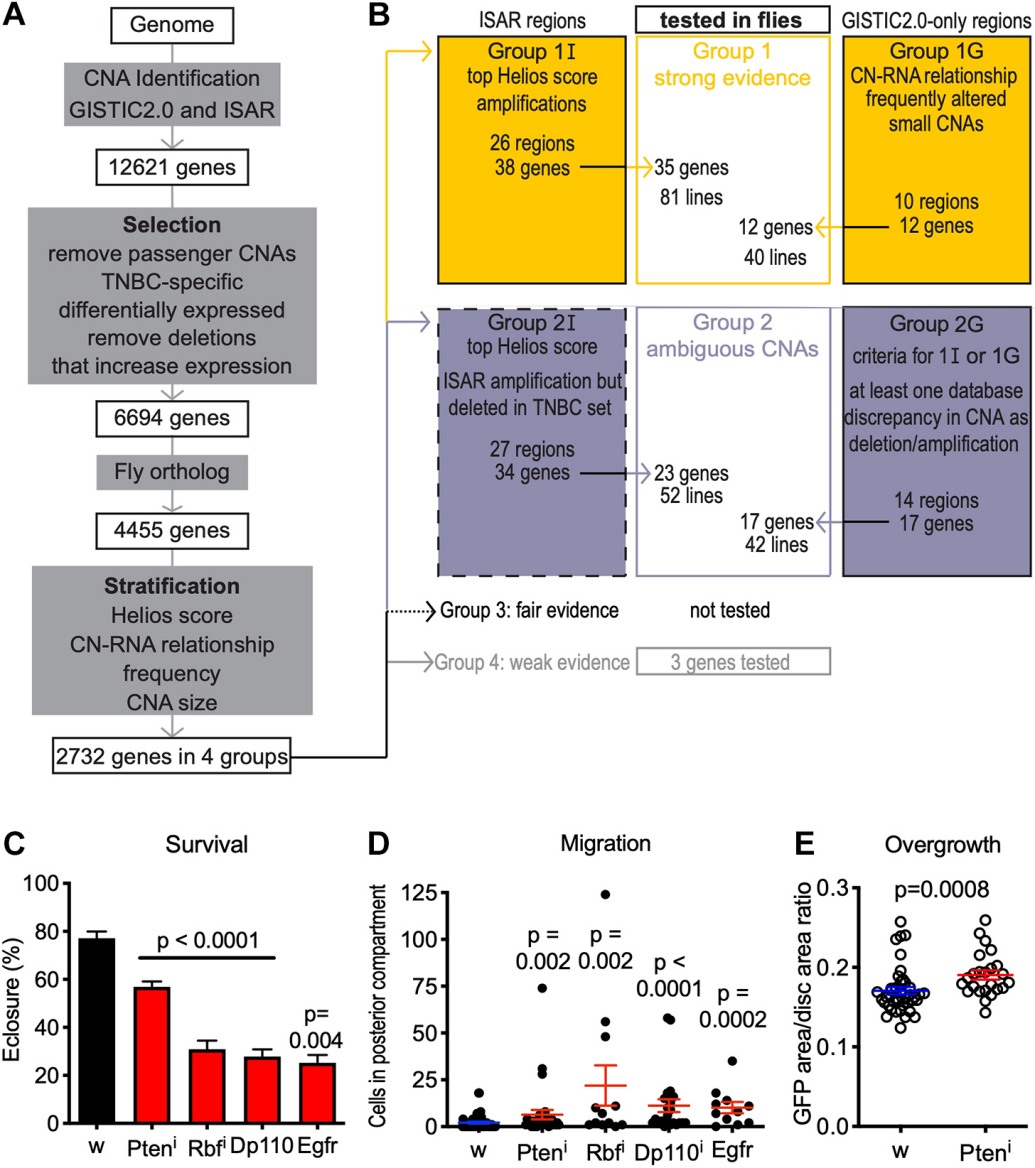
**Integrated computational-functional screen to assess potential TNBC driver genes.** (A) Prioritization scheme of potential driver genes from CNAs based on TCGA data. (B) Prioritized groups of genes for functional testing. Computational evidence is weaker for Group 2I (dashed border) than Group 2G. (C-E) Validation of the screening results: reduced activity after using RNA-interference (Pten^i^, Rbf^i^) or increased activity after overexpression (Dp110, Egfr). Four known driver genes *in trans* to *ptc>Myc,p53^sh^* led to decreased viability (C) (*n*=4 for Egfr, *n*=8 otherwise), increased cell translocation (D) and increased overgrowth of transgenic tissue (Pten^i^ shown as example in E) compared to *ptc>Myc,p53^sh^* alone. *P*-values reflect Student's *t*-test where data are normally distributed or, otherwise, Mann–Whitney test, compared to genotypically *white* (w) control flies (*ptc>w*). (w). See also Fig. S3 and Table S3.

Most of the 186 CNA regions identified by ISAR and GISTIC 2.0 contain dozens or hundreds of genes. Fig. 3A provides a flowchart to summarize our approach for prioritizing these genes for functional testing. To curate likely driver genes, we first eliminated genes that (i) occur in known, common copy number variants, (ii) were not differentially expressed, (iii) were associated with an increase in expression when copy number was reduced or, (iv) do not have a clear ortholog in *Drosophila*. To identify genes that are specifically relevant to TNBC, we analyzed the candidate genes for significance within the TNBC subset using a mild probabilistic cutoff. These steps reduced an original list of 12,621 candidates to 6694 (see Analysis of TCGA data in Materials and Methods; Fig. 3A).

When analyzing the TNBC dataset at this step, we found that some genes are deleted more frequently in TNBC than they are amplified, even though the CNA had been identified as an amplification by ISAR or GISTIC 2.0, and vice versa (see Analysis of TCGA data Materials and Methods). We examined these genes separately in functional testing (below) as Group 2. Indeed, several cancer genes have recently been found to have paradoxical, context-dependent roles (Walerych et al., 2012; Lobry et al., 2014; Shen et al., 2018). Because this study focuses on TNBC, we assigned each gene a CNA type according to our TNBC-specific analysis.

Of the 6694 genes with CNAs, 4455 had a clear fly ortholog, meaning they were available for functional testing. The genes were further stratified into groups based on: (i) the Helios score of each gene (for genes identified by ISAR) resulting from machine learning on TCGA and functional data in breast cancer (Sanchez-Garcia et al., 2014), (ii) whether copy number of a gene influenced its expression, (iii) whether the gene was altered more frequently than other genes in the region, and (iv) the size of the region (see Analysis of TCGA data Materials and Methods). Our analyses resulted in a set of prioritized genes for each common TNBC CNA (Table S2).

**Fig. 4.**
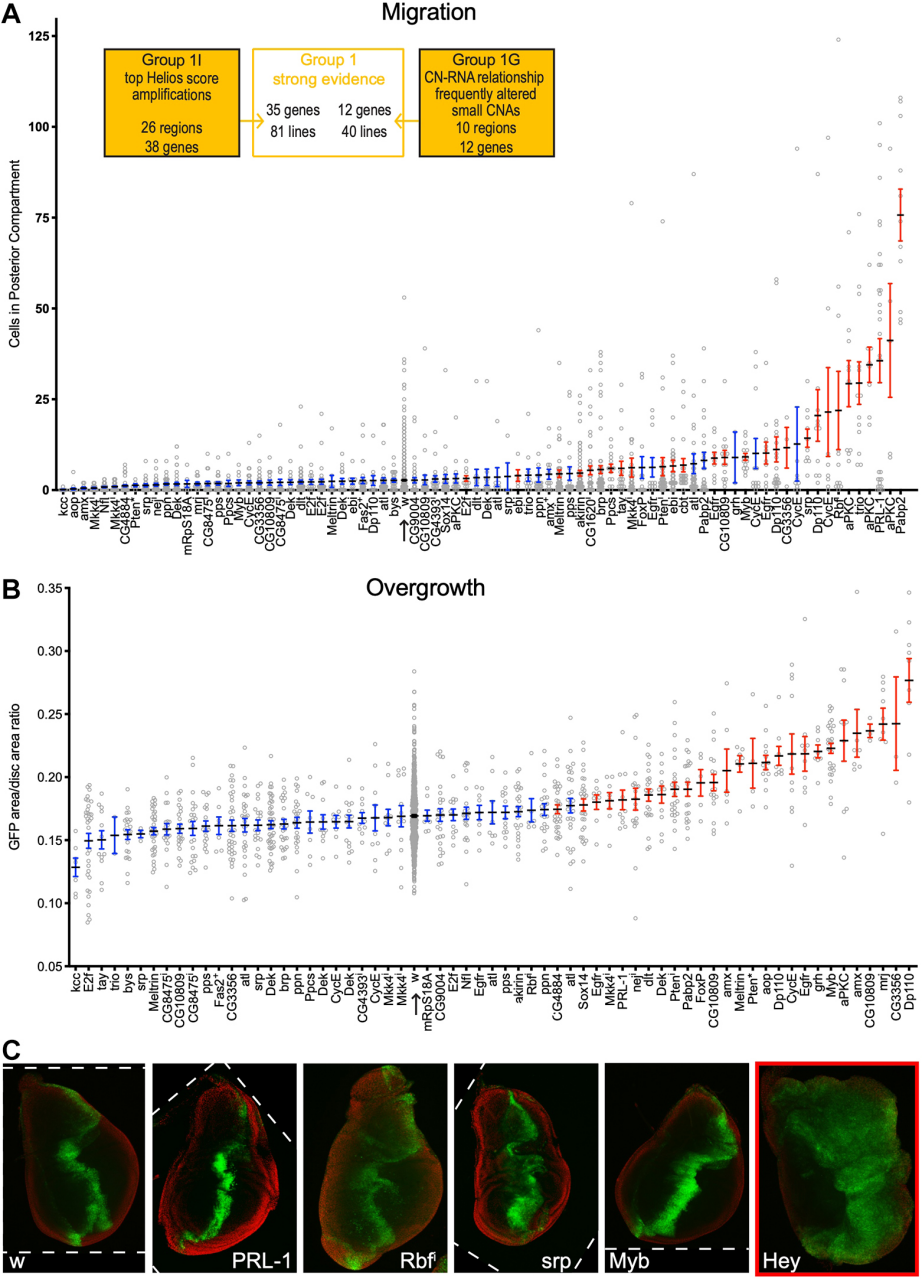
**Driver genes produce tissue phenotypes in the background of *Myc* and *p53^sh^*.** (A) Quantification of cell translocation for high-priority genes based on their known link to cancer progression. genes. Altering genes marked in red directed a significant increase in translocation compared to *ptc>w* controls (arrow), measured as *P*<0.05 in the original experiment and false discovery rate (fdr)<0.1 in the aggregate analysis shown here. Reducing activity of individual genes from each Group in the context of *ptc>Myc p53sh*, 16/52 from Group1I and 7/21 from Group 1G were significant. (B) Quantification of transgenic tissue overgrowth for high-priority genes. Altering genes marked in red directed a significant increase compared to *w* (arrow), measured as *P*<0.05 in the original experiment and fdr<0.1 in the aggregate analysis shown here. Some genes that are significant in this figure were not significant in their respective experiments due to variation between experiments. ^i^ indicates RNA-interference mediated knockdown; * indicates a heterozygous null allele;+indicates a duplication. 15/23 from Group1I and 5/12 from Group 1G were significant. (C) Selected phenotypes produced by specific driver genes: cell translocation (PRL-1, Rbf^i^, srp), small overt mass (Rbf^i^), disruption of morphology (srp), transgenic tissue overgrowth (Myb, Hey), and large overt mass (Hey), all compared to w. DAPI staining (red) highlights tissue boundary, GFP signal (green) demarcates transgene expression. Some images were rotated for comparison; borders are indicated by dashed lines. Translocation and overgrowth were not quantified for Hey (last image on right) because the large overt mass phenotype was 100% penetrant. In all cases, each gene was placed *in trans* to *ptc>Myc,p53^sh^* and compared to *ptc>Myc,p53^sh^* alone. See also Fig. S4 and Tables S12, S13.

For our functional studies, we focused first on the top ISAR genes (Group 1I; Fig. 3A), small GISTIC 2.0 regions (Group 1G; Fig. 4A), select genes with ambiguous classification of CNAs (Group 2), and three lower ranked genes for which we had fly lines on hand (Group 4G). We then tested *Drosophila* lines representing these genes in functional assays, in many cases testing multiple lines for each gene (Fig. S3A). Control lines included three with transgenes unrelated to human genes, and one p53 null allele – expected to have no effect when p53 was knocked down using RNA interference (RNAi). In general, we determined how to analyze genes based on their status in our TNBC-specific CNA analysis: genes comprising deletions were treated as tumor suppressors, whereas those comprising amplifications were treated as oncogenes.

### Driver genes enhance p53/Myc transformation phenotypes

Our computational analysis and ranking protocol identified a set of candidate driver genes within TNBC-associated regions of CNA. To functionally assess candidate genes, we placed candidate transgenes *in trans* to *ptc>Myc,p53^sh^*, creating a ‘3-hit’ model by standard genetic crossing. Amplified genes were assessed by overexpression (*ptc>Myc,p53^sh^/UAS-candidate*); deleted genes were assessed by RNAi-mediated knockdown (*ptc>Myc,p53^sh^/UAS-RNAi[candidate]*) or by removing one functional copy (*ptc>Myc,p53^sh^/mutant^−/+^*).

To validate our approach, we tested four well-known tumor drivers: oncogenes Dp110 (officially known as *Pi3K92E*) and Egfr were overexpressed, and tumor suppressors Pten and Rb were reduced by knockdown in the context of *ptc>Myc,p53^sh^*. In each case (4/4), adding the cancer-associated gene to a *ptc>Myc,p53^sh^* background decreased survival to adulthood (eclosion rate; see Lethality analyses in Materials and Methods; Fig. 3C); adding Pten also decreased larval survival (pupariation rate; Fig. S3B). These genes also significantly increased translocation of cells within the wing disc (Fig. 3D) compared to *ptc>Myc,p53^sh^* alone. For Pten, an example of a driver that showed only mild translocation and overgrowth of transgenic tissue was significantly increased (Fig. 3E). In contrast, 0/4 Group 4 lines and 1/4 control lines showed significant reduction in eclosion (Fig. S3C). Three lines of Group 4 and three control lines showed significant reduction in pupariation, perhaps due to variation in genetic background (Fig. S3D). However, 0/4 Group 4 lines and 0/4 control lines showed significant change in cell translocation (Fig. S3E). Furthermore, 0/3 Group 4 lines and 0/2 control lines showed significant increase in transgenic tissue overgrowth (Fig. S3F). In summary, well-known drivers enhanced aspects of transformation in the *ptc>Myc,p53^sh^* model while non-drivers did not.

As a first step in identifying functional driver genes within regions of CNA, we functionally tested 222 fly lines, covering 47 of the 50 genes in Group 1, 40 of the 53 genes in Group 2, three of 2023 genes in Group 4G, and three control genes. Of the 222 lines tested over all groups, 100 exhibited increased lethality when placed in *trans* to *ptc>Myc,p53^sh^*, compared to *ptc>Myc,p53^sh^* alone, defining 69 separate genes (Tables S4, S9, S10)*.* To determine whether genes that decreased survival also increase aspects of transformation, we tested 66 of these genes in a *ptc>Myc,p53^sh^* compared to *ptc>Myc,p53^sh^* alone (multiple lines in some cases; Tables S4, S8-S10) for changes in cell translocation. For genes that did not show a significant change in translocation in an initial trial, we also measured tissue overgrowth. Together, 48/66 genes (63/116 lines) – when placed in a *ptc>Myc,p53^sh^* background – significantly increased translocation (Fig. 4A,C and Fig. S4A; Table S12) or overgrowth (Fig. 4B,C and Fig. S4B; Table S13) phenotypes; these 48 genes were judged to be functional drivers in this context.

We identified some differences in the percentage of drivers identified (hereafter referred to as ‘hit rate’) within each computationally defined group. Most tested Group 1 genes were drivers, including 56% of genes in Group 1I and 75% of genes in Group 1G. The higher hit rate for Group 1G suggests that genes in small GISTIC 2.0 regions are especially likely to be regulators of tumor progression (‘hereafter referred to as ‘drivers’). In Group 2G, where listings within at least one database were in agreement with our TNBC-specific analysis for each gene, 76% genes were drivers, a similarly high hit rate compared to that in Group 1G. However, only 38% genes tested in Group 2I – i.e. regions identified as amplifications by ISAR but deletions by our TNBC-specific analysis – were drivers. This suggests that algorithms, such as GISTIC 2.0 and ISAR are useful for identifying functional amplifications and deletions. That is, genes appearing in regions identified only as amplifications are generally unlikely to function as tumor suppressors. We opted not to test the remainder of the genes in Group 2I.

**Fig. 5.**
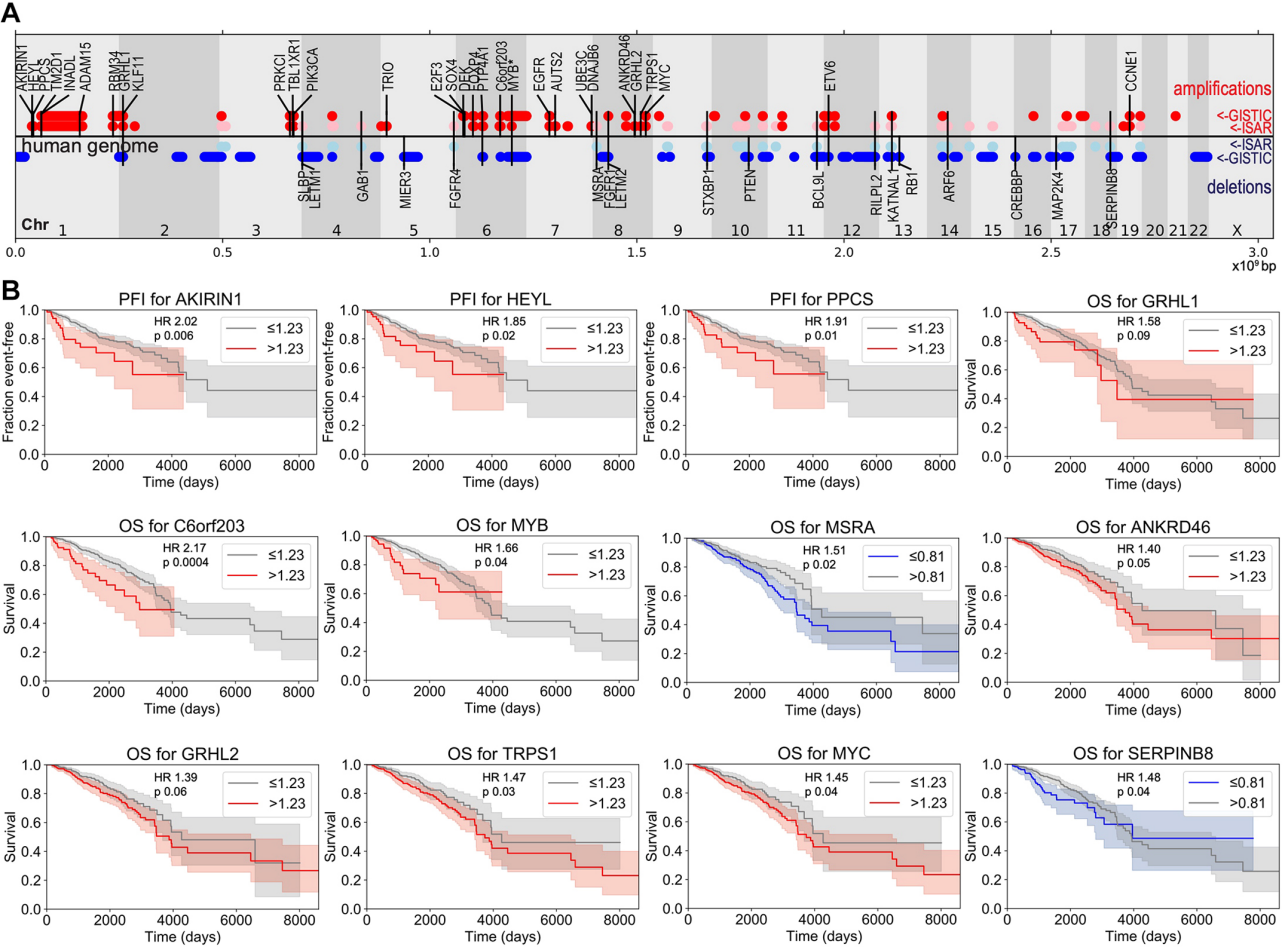
**Known and novel driver genes in TNBC identified functionally.** (A) Map of genomic regions that are amplified (red) and deleted (blue) in TNBC, and the location of functionally validated TNBC driver genes identified in our screen. Group 2I regions, representing some ambiguity, are represented in pink and light blue. Genes comprising an ambiguous CNA type are represented with a line extending through both amplified and deleted regions. Genes above the horizontal axis are oncogenes; genes below the axis are tumor suppressors. MYB (indicated by *) can function as both. (B) Kaplan–Meier curves of progression-free interval (PFI) or overall survival (OS) in the TCGA breast cancer dataset for CNA driver genes (log-rank *P*-value<0.1). Amplified genes in red; deleted genes in blue. HR=Cox hazard ratio. See also Tables S1, S5 and S7. Similar-appearing Kaplan–Meier curves for different genes reflect genes from the same CNA region that are likely to be altered in the same cohort of patients (see Discussion).

Altogether, in addition to *MYC* our analyses identified 48 identified drivers. These 49 genes define a functional set of CNA-associated putative driver genes (Fig. 5A; Table S1) and account for the observed copy number aberration of 66 partially overlapping, computationally defined regions (Table S5). Of note, several of these driver genes have been reported in *in silico* or *in vitro* screens for drivers (Tables S1 and S6) (Ciriello et al., 2013; Aure et al., 2013; Vogelstein et al., 2013; Forbes et al., 2015; Marcotte et al., 2016; Griffith et al., 2017). Furthermore, four of the genes were determined to have been mutated at a significant rate (Table S2). Finally, we assessed these genes for effects on survival in the TCGA breast cancer dataset (Liu et al., 2018). Twelve genes showed a trend towards increased progression or decreased survival (Fig. 5B) in patients (Table S7; see Lethality analyses Materials and Methods). Most (31/49) of these genes showed evidence of confirmation of the driver status when using at least one of these methods (Table S1). This analysis demonstrates the strength of our computation/genetics approach to identifying functional drivers.

### Genetic complexity abrogated drug response in TNBC models

Work by a variety of laboratories including ours suggest that one source of resistance to therapeutic drugs is genetic complexity of the tumor. To test the effects of genetic complexity on drug response, we used our database of candidate CNA-associated driver genes to generate six 3-hit models that reflect some of the genetic complexity in TNBC. Specifically, we paired *ptc>Myc,p53^sh^* with overexpression of oncogenes Dp110, Hey, Myb, Ppcs or aPKC, or knockdown of the tumor suppressor Rop.

**Fig. 6.**
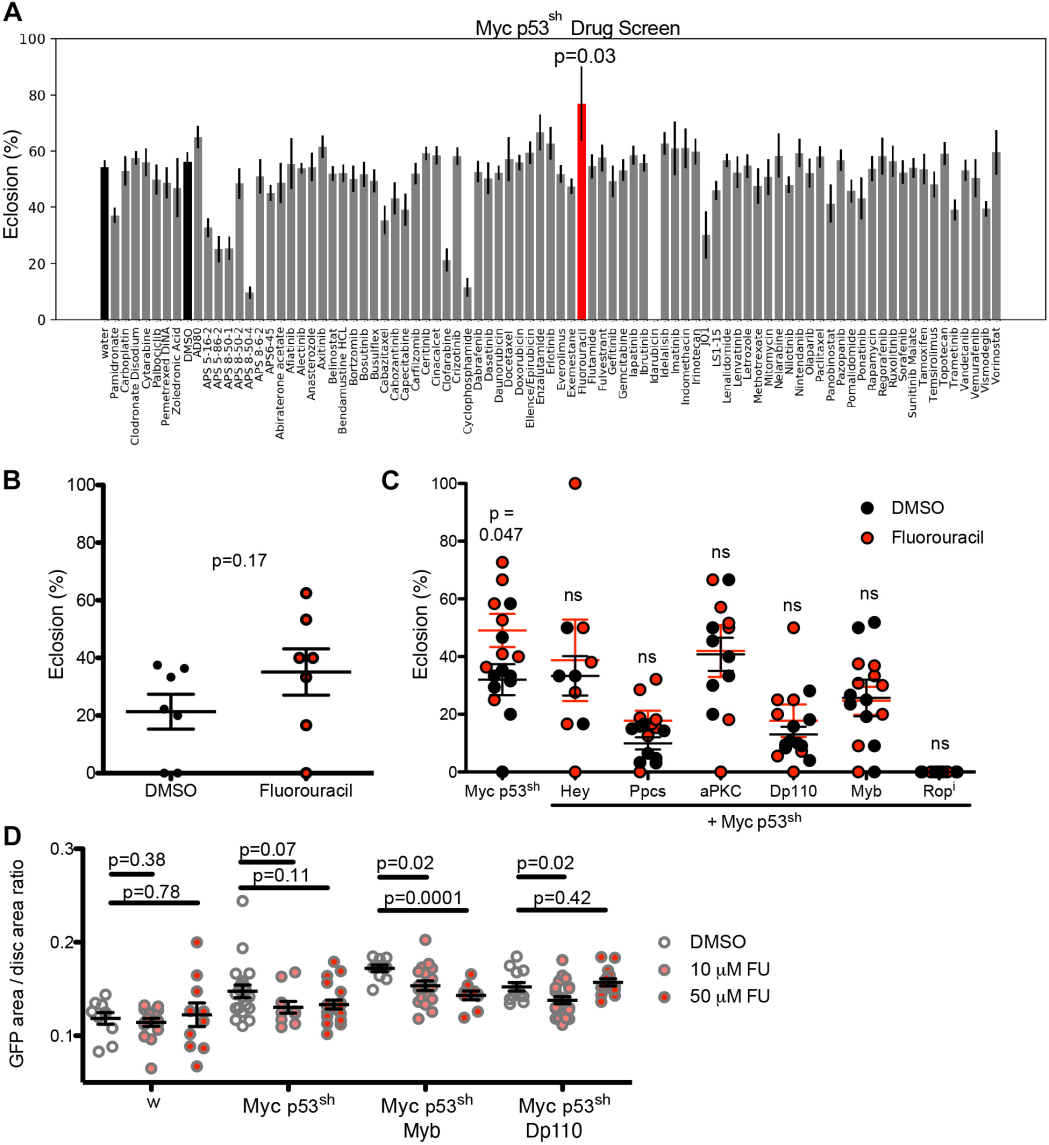
**Genetic modifiers abrogate the response of *p53^sh^ Myc* to fluorouracil.** (A) *ptc>Myc,p53^sh^ Drosophila* strains were cultured in medium containing screening-optimized doses of cancer drugs at 27°C. Viability was assessed and eclosion rate for each drug is shown in percent. DMSO was used as a control for drugs dissolved in DMSO and water was used as a control for drugs dissolved in water (black bars). Fluorouracil (red bar) significantly improved viability (Mann–Whitney *U* test versus DMSO: *P*=0.03). (B,C) Fluorouracil was tested on the ptc>Myc,p53^sh^ line at 27°C (B) and the ptc>Myc,p53^sh^ line plus six selected driver genes (Hey, Ppcs, aPKC, Dp110, Myb, Rop as indicated) at 27°C (C). In each case, addition of an additional driver led to loss of fluorouracil-mediated rescue. ns, not significant. (D) Fluorouracil was tested at two doses (10 or 50 µM) on control (*ptc>w*), *ptc>Myc,p53^sh^* (Myc p53^sh^), *ptc>Myc,p53^sh^,Myb* (Myc,p53^sh^ Myb) and *ptc>Myc,p53^sh^,Dp110* (Myc,p53^sh^ Dp11) flies, and transgenic tissue overgrowth was quantified as described in Fig. 4B. Two-way ANOVA results were (C) genotype: *P*<0.0001, drug: ns, interaction: ns; (D) genotype: *P*<0.0001, drug: *P*=0.0054, interaction: *P*=0.0883. Displayed *P*-values reflect *t*-tests (see Materials and Methods). See also Table S8.

To test the effect of genetic complexity on drug response, we screened 72 FDA-approved cancer drugs, JQ1 (targeting Myc-pathway activity), and five novel drugs that have shown activity in other *Drosophila* cancer models (Fig. 6A). Drugs were tested at 27°C to provide an optimal level of lethality, allowing us to use rescue as a quantitative assay. Drugs were mixed into the culture medium of *ptc>Myc,p53^sh^* flies to be consumed orally. Fluorouracil, a chemotherapy drug used in the treatment of TNBC (Muss et al., 2009), provided the strongest rescue of *ptc>Myc,p53^sh^*-induced lethality at both 27°C (Fig. 6A,B) and 29°C (Fig. S5A), mirroring activity in patients with TNBC. In contrast, all six selected 3-hit TNBC lines built from our functional database failed to show significant rescue by fluorouracil at either 27°C (Fig. 6C) or 29°C (Fig. S5B).

We examined the impact of fluorouracil on transgenic tissue overgrowth on two selected 3-hit lines. *ptc>Myc,p53^sh^* showed a trend toward decreased overgrowth in response to fluorouracil (10 or 50 µM; Fig. 6D), consistent with rescue from lethality (Fig. 6A-C). The response was attenuated in response to overexpression of Dp110, such that no response to 50 µM was observed. The addition of Myb produced a strong dose-response in reduction of tissue overgrowth, yet notably, this did not translate into whole-animal rescue (Fig. 6C). We conclude that, similar to other tumor types (Bangi et al., 2016), increased genetic complexity can lead to drug resistance in models of TNBC.

## DISCUSSION

Producing useful genetic models of cancer and designing targeted therapies require an understanding of the genes that drive tumor progression. This is especially challenging with TNBC, a disease in which most driver genes emerge from copy number aberration rather than mutation, and carry many passengers with them. Approaches to identifying driver genes in this context have included statistical analyses of breast tumor-sequencing data (Aure et al., 2013), pan-cancer analyses (Ciriello et al., 2013; Vogelstein et al., 2013; Forbes et al., 2015), crowdsourcing (Griffith et al., 2017), machine learning (Sanchez-Garcia et al., 2014; Schroeder et al., 2014), cell culture screening approaches in transgenic lines (Kessler et al., 2012) and breast cancer cell lines (Marcotte et al., 2016; Koedoot et al., 2019; Patel et al., 2018), and a recent forward-genetics approach in a mouse model of BRCA1-deficient TNBC (Miao et al., 2020). Our study complements this body of literature by providing a systematic characterization of putative TNBC driver genes in a whole animal model, using a genetic background representative of a majority of patients with TNBC.

Our data are consistent with previous evidence, i.e. that mutation of TP53 (Cancer Genome Atlas Network, 2012; Jiang et al., 2019; Nagahashi et al., 2018) and amplification of MYC (Cancer Genome Atlas Network, 2012; Jiang et al., 2019; Curtis et al., 2012) are the two most common genetic aberrations in TNBC, and frequently occur in combination (Fig. 1). In *Drosophila*, Myc promoted aspects of transformation including tissue overgrowth and cell translocation (Fig. 3). Knockdown of p53 enhanced Myc lethality but not overgrowth or translocation (Fig. 2). These data indicate that p53 is likely to affect other processes in the targeted tissue, such as senescence or metabolism, which can impact survival in *Drosophila* (Bangi et al., 2016; de la Cova et al., 2014). Our ‘base’, i.e. the *Myc,p53^sh^* transgenic line, constitutes a simple genetic animal model of TNBC, exhibiting activation of matrix metalloprotease and caspase cleavage, previously validated markers of cell translocation and metastasis-like behavior in fly cancer models (Levinson and Cagan, 2016; Vidal et al., 2006; Sonoshita et al., 2018).

Many fly lines showed increased lethality when placed *in trans* to *ptc>Myc,p53^sh^*, including all known cancer drivers tested and several negative controls (Fig. S3C,D), suggesting that rescue of lethality provides a sensitive but not specific assay. However, only altering cancer-related genes – increasing ortholog expression of oncogenes or decreasing activity of tumor suppressors – altered transformation phenotypes in the *Drosophila* wing disc, providing a more-specific second-line assay. Based on these functional assays, we identified or confirmed the cancer driver activity of 49 genes (Fig. 5A). In some cases, multiple driver genes are present within the same CNA region (Table S1). This phenomenon was suggested by computational predictions, such as Helios (Sanchez-Garcia et al., 2014), and confirmed in our functional experiments. The impact of multiple driver genes in the same amplification or deletion, including whether those genes interact, remains an area open for investigation. We should also note that genes without clear *Drosophila* orthologs were not tested using our approach.

Some drivers, such as *CCNE1*, are well-established cancer drivers. Others, such as *TRIO*, are less well-studied but have previously appeared in the literature and databases of cancer driving genes. *DNAJB6* has the highest Helios score in its region but is not considered a driver based in an assay of anchorage-independent growth (Sanchez-Garcia et al., 2014). Its observed effects in a translocation assay (Koedoot et al., 2019) and our overgrowth assay highlight the importance of testing for drivers in multiple contexts, such as an intact epithelium, in which competition with wild-type tissue can be observed. To our knowledge, *PPCS*, *TM2D1*, *INADL*, *RBM34*, *C6orf203* and *STXBP1* have not been linked to breast cancer. The variety of driver genes identified in this study underscores the potential of an integrated computational/experimental approach.

The high hit rates in Groups 1 and 2G (57-76%) indicate a significant enrichment in driver genes based on computational work alone. None of the Group 4 genes tested were identified as drivers. This suggests computational features can enrich for driver genes over the initial identification of CNA regions by GISTIC 2.0 and ISAR. Some genes exhibited paradoxical properties. A small number of genes were amplified in the TCGA data but demonstrated decreased expression. For one such gene, *MYB*, we tested for both tumor suppressor and oncogene activity, and found that knockdown led to increased cell translocation (Fig. 4A), while overexpression led to tissue overgrowth (Fig. 4B). Similarly, our study helped deconvolve genes in ‘ambiguous’ Group 2 CNA regions. For genes identified as amplified in one TNBC database and deleted in another, 76% exhibited driver effects when tested according to whichever CNA occurred more frequently in TNBC.

In contrast, genes in regions only identified as amplifications by using ISAR but more frequently deleted in the TNBC set (Group 2I) were less likely to be drivers (38% hit rate). Our assays demonstrated that the driver genes identified in this group function as tumor suppressors, despite appearing in amplified regions (Table S10). One example, *MSRA*, has been found to be a tumor suppressor in other functional studies (Lei et al., 2007; Luca et al., 2010). Together, these data suggest that, when altered by different CNAs, the same gene can become either an oncogene or a tumor suppressor, depending on subtype and biological context.

Developing a functional database allowed us to then ask a question significant to the development of therapeutics: Does genetic complexity alter drug response, when the ‘base’ set of common drivers is the same? We found that, while fluorouracil rescued our *Myc,p53^sh^* base mode from lethality and mitigated transgenic tissue overgrowth, introducing an additional transgene variously enhanced or prevented the effect of fluorouracil on tissue overgrowth in a dose- and gene-dependent manner. Yet in all cases tested – including examples of both oncogenes and tumor suppressors, and genes from both Group 1 and 2 – the additional transgene abrogated fluorouracil-induced rescue from lethality. This suggests that increasing genetic complexity reduces treatment response, confirming findings from work in other tumor models (Bangi et al., 2016). Our data indicate that genetic complexity may play a role in the poor outcomes for patients with TNBC (Dent et al., 2007), and suggest genes that are candidates to mediate chemoresistance. Our system also provides a platform to study the combinatorial effect of genetic aberrations on multiple tumor-like phenotypes. In the future, this system could be used to examine the mechanisms by which specific genetic combinations confer resistance to therapy.

Using a functional approach, we have identified multiple new driver genes that help explain specific amplifications and deletions found in patients with TNBC. Some of these genes are recently discovered or poorly understood cancer drivers that merit further research into the specific roles they play in TNBC. Further, we provide data that some of these genes can mitigate the response of a *Myc,p53^sh^* model to the chemotherapeutic fluorouracil. Further understanding the functional impact of these driver genes on drug response may help guide prognosis and drug selection based on genotype, and suggest new avenues for therapeutic development.

## MATERIALS AND METHODS

### Analysis of TCGA data

Data from the invasive breast carcinoma dataset were downloaded from The Cancer Genomic Atlas (TCGA) data portal (National Cancer Institute, 2018, https://www.cancer.gov/ccg/research/genome-sequencing/tcga ). The dataset contains 1100 cases.

The somatic mutation annotation file (MAF) for this dataset contains 771 cases and 14,375 genes. We uploaded this file to the MutSigCV public server (Lawrence et al., 2013) to retrieve predicted mutated driver genes for breast cancer. According to Lawrence et al. (2013), genes with q<0.1 were considered significant. We also considered genes from COSMIC (Forbes et al., 2015), the TCGA Pan-Cancer Analysis (Ciriello et al., 2013), the Vogelstein dataset (Vogelstein et al., 2013) and CIViC as of April 2016 (Griffith et al., 2017) to be driver genes that might be mutated. Mutations in any of these genes are shown in Fig. 1A. Known predisposing germline variants were found in 52 cases (Cancer Genome Atlas Network, 2012). Mutations in any of these ten genes were included in Fig. 1A.

The level 3 copy number SNP array was downloaded for all 1020 samples for which it was available. Candidate genes included those reported by GISTIC 2.0 (total amp, basal amp, total del and basal del datasets) (Cancer Genome Atlas Network, 2012) and ISAR (from the total dataset and the basal dataset, defined by the authors as ER/progesterone receptor negative) (Sanchez-Garcia et al., 2014), resulting in 12,621 genes. For each of these, any gene symbol synonyms were converted to a consensus gene symbol from HUGO (Braschi et al., 2019). For each of these genes, coordinates were retrieved from NCBI build GRCh37 (also known as hg19). Raw copy number values were retrieved from the SNP array files labeled ‘no_cnv’, in which germline copy number variants known to occur in the population had been removed by TCGA. At this step, 541 genes were removed from the candidate list because they could not be identified due to nomenclature or missing data, and 51 were removed because they only appeared in known germline copy number variants not included in the ‘no_cnv’ files. Whenever a value in the SNP array, in which the data are on a log_2_ scale, represented a region covering the entire gene, the copy number for that gene was retrieved and converted to a linear scale:

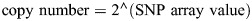


This value was then adjusted according to an estimate of tumor purity [the fraction of the sample that is tumor (Aran et al., 2017)]. To account for germline variations in copy number, a fold change over the germline copy number was calculated, giving this formula for the final adjusted copy number:

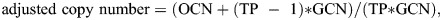


where OCN=observed copy number in the primary tumor sample; GCN=germline copy number; and TP=tumor purity.

In the minority of cases where a tumor-purity estimate or germline copy number was unavailable, a value of 1 was substituted for each of these parameters.

Thus, an adjusted copy number value of 1 represents normal in this study. Similar to other studies of CNAs (Aure et al., 2013; Akavia et al., 2010), cutoffs of 2^0.3 and 2^-0.3 were used for amplifications and deletions, respectively, representing the gain and loss, respectively, of one copy in ∼40% of the tumor. The copy number for each region shown in Fig. 1A represents the average of all genes in the region. The 72 TNBC cases with both copy number data and somatic mutation data are shown in Fig. 1A.

Neither GISTIC 2.0 nor ISAR was applied to this dataset with TNBC specifically in mind. Thus, following these analyses, genes irrelevant to TNBC biology might appear in CNA regions, such as the estrogen receptor 1-encoding gene *ESR1*. GISTIC 2.0 was applied to the basal-like gene expression subtype, which only partially overlaps with TNBC. ISAR was applied to the ER/progesterone receptor-negative subgroup the authors refer to as ‘basal’, although this would also include CNAs that are relevant to HER2-positive tumors. These partially overlapping datasets resulted in some discrepancies. Some regions are referred to as amplifications by GISTIC 2.0 in the total breast cancer set but as deletions in the basal subtype set. Similar discrepancies also appeared between the ISAR and GISTIC 2.0 results (and were referred to as ‘ambiguous CNAs’ in this current study). As ISAR has been explicitly designed to pick up amplifications even when they appear in the context of a larger deletion (Sanchez-Garcia et al., 2014), this might explain some of the discrepancies.

To select genes within CNA regions that are specifically relevant to TNBC biology and to determine the direction of their effect, we applied an additional filter. Following the study by Aure et al. (2013), for each gene, we calculated the binomial probability of seeing the number of TNBC tumors with an amplification or deletion of that gene that appear, out of the 110 TNBC tumors in the dataset. We retained genes with a probability <0.05 for further analysis. Since this step represents an extra filter on top of an already rigorous algorithm to detect CNAs, we did not use multiple hypothesis correction. By using this filter, 2321 genes were removed. If more deletions of the gene appeared in the dataset than amplifications, we marked the gene as a deleted and treated it as a putative tumor suppressor; when more amplifications than deletions appeared, we treated it as amplified and a putative oncogene.

Next, we required that candidate genes within CNA regions be differentially expressed. We downloaded the RNA-seq data for all samples available and extracted the transcript quantification values for each gene, which had been generated by TCGA using RSEM (Li and Dewey, 2011; Ciriello et. al, 2015); 2693 genes did not have RNA data available and so were removed at this step. As described by Akavia et al. (2010), we retained genes with a standard deviation of greater than 0.25 for further analysis, thus removing 306 genes. We then converted the transcript quantification values to z-scores for each gene. For each amplified gene, we performed a statistical test comparing the z-scores of primary tumors with that amplification and those without. We performed the equivalent analysis for deleted genes. Where the two distributions had equal variance, we used a Student's *t*-test, and where they did not, we used the Welch’s test. All distributions were either normally distributed or had a sample size of ≥30, so we judged parametric statistics to be valid. We then applied the Bonferroni correction to all resulting *P*-values.

For further analysis, we retained genes whose copy number had a direct positive relationship or a non-significant relationship with expression. Because amplifications could conceivably cause chromatin changes that reduce the expression of a tumor suppressor, we also retained genes that had reduced expression when amplified. However, as no theoretical mechanism could cause increased expression of a cancer driver gene when it is deleted, we removed genes that were associated with increased expression when deleted from further analysis (15 genes).

For genes appearing in the ISAR dataset, we selected the top 1-3 genes by Helios score for Group 1I (Table S3). The remaining genes in the top 3 (for regions with 12 or fewer genes) or top quartile (for regions with more than 12 genes) of Helios scores made up tier 2. Because the Helios score is based in part on the frequency of CNAs in the dataset as well as the relationship between copy number and expression, we did not directly consider these parameters to define Group 1I.

From the GISTIC 2.0 genes that did not appear in the ISAR data, we selected genes that 1) have a significant, positive relationship between copy number and expression, 2) appear in the top 3 (for regions with 12 or fewer genes) or top quartile (for regions with more than 12 genes) of genes in the region by the frequency of amplification or deletion occurring in the dataset, and 3) occurred in small (<10 genes) regions. These genes comprise Group 1G (Table S3).

Genes marked for inclusion in Group 1 were moved to Group 2 when there were discrepancies between databases. In Group 2G, each gene belongs to at least one region identified by GISTIC 2.0, and some also belong to amplifications identified by ISAR. Genes in this group belong to at least one amplification and at least one deletion, and were analyzed as tumor suppressors or oncogenes according to the result of our TNBC-specific analysis. In Group 1I, genes belong to amplifications in ISAR but appeared to be deleted more frequently in our TNBC-specific analysis. Select genes in this group were analyzed as tumor suppressors. The remaining ISAR and GISTIC 2.0 genes comprise Group 3 and 4 (Fig. 3B).

Finally, we converted all human genes in these five tiers to fly genes by using homologs compiled from the Drosophila Interactions Database (DroID) (Murali et al., 2011; downloaded 1/2014); DRSC Integrative Ortholog Prediction Tool (DIOPT) (Hu et al., 2011; downloaded 10/2014), HomoloGene (https://www.ncbi.nlm.nih.gov/homologene), Ensembl (Flicek et al., 2014) and OrthoDB (Waterhouse et al., 2013) (the latter three downloaded 7/2014). For genes with multiple possible fly orthologs, we performed the search manually in DIOPT and performed a tBLASTn (Mount, 2007; Johnson et al., 2008) search of the human protein sequence against the fly genome. The top scoring orthologs from each of these methods were used for testing (Table S11).

### Fly stocks

Experiments with overexpression of Myc used stock #9675 (Bloomington Drosophila Stock Center) with genotype hs-FLP y w; UAS-Myc. Initial experiments with p53 knockdown used a long hairpin under upstream activating sequence (UAS) control VDRC Id 38235 (Vienna *Drosophila* Resource Center). This is represented as *p53^lh^*. However, although the long hairpin siRNA produced an effective knockdown, it also produced a faster migrating species on western blots, possibly representing expression of a different P53 isoform. Due to this and the convenience of using a hairpin on the third chromosome, a short hairpin with guide sequence 5′-TGCTGAAGCAATAACCACCGA-3′ under UAS control on the third chromosome, generated in our lab, was used. We generated a recombinant third chromosome with this *UAS-p53^sh^* and *UAS-Myc*. This line has the genotype *w; UAS-Myc UAS-p53^sh^*; flies in Fig. 1B are in a yellow hairy singed forked (yhsf) background, chromosome markers that should not affect the transgene phenotypes. Both hairpins produce an ∼80% knockdown in the presence of *UAS-Myc*, but *p53^sh^* does not exhibit the shift in band size (Fig. S2B).

In initial lethality experiments crossing *Myc p53^sh^* to *ptc-Gal4*, low numbers of progeny resulted in survival variability after the first two technical replicates (flips). Therefore, only the first two technical replicates from each of seven different independent experiments (14 replicates total) were included in the analysis shown in Fig. 1C.

We combined this line with patched-Gal4 on the second chromosome, the linked balancer *SM5(Gal80)-TM6B*, and a *hs-hid* construct on the Y chromosome to generate *(hs-FLP) (y) w /hs-hid; (ptc-Gal4; UAS-Myc UAS-p53^sh^)/SM5(Gal80)-TM6B*. This line was used for lethality experiments. We also generated a version with *UAS-GFP* on the second chromosome. *hs-FLP* was not detected in a PCR of fly genomic DNA from >30 flies from this line, indicating it likely lost the floating *hs-FLP* construct on the X chromosome, and so has genotype *(y) w /hs-hid; ptc-Gal4 UAS-GFP; UAS-Myc UAS-p53^sh^/SM5(Gal80)-TM6B.* This line was used for the overgrowth and cell translocation assays.

To generate virgins of both these lines, bottles were incubated for 1-3 h at 37°C twice for 1-5 days after lay, activating the hid protein and resulting in death of all fertile males before eclosion. Virgins from heat shocks were only used for experiments and never put back into the stock.

Genotypes and stock numbers for lines used in the genetic screen are listed in Tables S9, S10. Genotypes and stock numbers related to Fig. 4A,B and Fig. S4 are in Tables S12, S13. Stock numbers related to the genotypes shown in additional figures are as follows, in order (exclusive of *w*):

Fig. 3C-E and Fig. S3B: VDRC35731, VDRC10696, in house UAS-Dp110 stock, BL9532

Fig. S3D (and related panels): BL20108, DGRC203601, BL17396, BL14265

Fig. S3E (and related panels): BL6659, BL6660, BL33623, BL6815

Fig. 4C: BL20713, VDRC10696, d10248, F001574, F000566.

### Western blotting

*765-Gal4* was crossed to lines carrying p53 knockdown or *UAS-Myc* to express the transgenes in the entire wing disc. Wing discs from L3 larvae were dissected in cold PBS. Ten discs were placed in RIPA buffer with protease inhibitor and phosphatase inhibitor. LDS sample buffer was added, and the mixture was heated to 70°C for 5 min, then frozen at −80°C until further use. Samples were run on a Thermo Fisher 4–12% bis-tris NuPAGE gel as per manufacturer’s instructions. Protein was transferred onto a PVDF membrane, which was then probed against p53 (DSHB p53-H3 s 1:1000), syntaxin (DSHB 8B-3, 1:1000) or Myc (Santa Cruz Myc d1-717, 1:200) overnight at 4°C. Detection was performed with the Thermo Fisher Pierce™ ECL Western Blotting Substrate as per manufacturer’s instructions. Quantification was performed in ImageJ using the Gel Analyzer tool.

### Lethality analyses

To measure survival with two or fewer genes of interest, *ptc-Gal4* was crossed to the genotype of interest (i.e. *UAS-Myc UAS-p53^sh^*) and the percentage eclosion rate was calculated as: 100×[number of empty pupal cases/(number of uneclosed pupae+number of empty pupal cases)]. Crosses for the genetic screen were set up at 25°C as *(hs-FLP) (y) w*/*hs-hid; ptc-Gal4; UAS-Myc UAS-p53^sh^*/*SM5(Gal80)-TM6B *×*UAS-gene* or *UAS-gene*/*FM7c 2xTb-RFP* or *UAS-gene*/*CyO 2xTb-RFP*, or *UAS-gene*/*TM6B*.

For genes of interest that were homozygous, or heterozygous on the first, second or third chromosomes, respectively (Pina and Pignoni, 2012). For second and third chromosome genes of interest, males of the stock were used and virgins of the stock we generated as described above.

Because chromosomes of the *SM5(Gal80)-TM6B* balancer are genetically linked by a reciprocal translocation, all progeny in the cross had either *ptc-Gal4; UAS-Myc UAS-p53^sh^* or the balancer, which produces a tubby phenotype. Similarly, because of the balancers used, all progeny that did not have the gene of interest had a tubby phenotype. Eclosion was calculated as above only for the non-tubby pupae. Where the gene of interest was homozygous, a relative pupariation rate was also calculated as 100×[number of *non-tubby* pupae/number of tubby pupae]. This was not calculated where the gene of interest was homozygous lethal and used with a balancer.

To account for the effect of variation in the genetic background of the stocks on survival, two biological replicates were set up for each gene of interest in each experiment. These were flipped four times, for a total of eight replicates for each genotype. Eclosion and relative pupariation for each gene of interest were compared to control with a Student's *t*-test where distributions were normal or a Mann–Whitney u test where distributions were not normal. When either eclosion or relative pupariation was lower than control (*P*<0.05), the experiment was repeated. Genes that significantly lowered lethality in at least two independent experiments were considered candidates for tissue phenotype analysis. For simplicity, the aggregate of repeat experiments is shown in Fig. 3C and Fig. S3B-D.

### Wing disc analyses

To visualize transformed tissue, we performed the equivalent crosses at 25°C as described above with a UAS-GFP construct on the second chromosome. Wing discs from non-tubby L3 larvae were dissected, fixed, and mounted in Vectashield with DAPI. Apical-basal and anterior-posterior axes were determined by locating the patched-expressing region of the peripodial membrane (Wu et al., 2004). Cleaved caspase and matrix metalloprotease were visualized by immunostaining the fixed tissue (Cell Signaling antibody #9661, 1:200; DSHB antibody 3B8D12, 1:10). To assess translocation, the discs were visualized at 40× magnification on a Leica DM5500Q confocal microscope and the number of GFP-expressing cells in the posterior compartment was counted.

Genes of interest that did not have a significant effect on cell translocation were tested with the more laborious overgrowth assay. Each disc was imaged at 10× magnification, using identical settings within each imaging session, and the red and green channels were exported to separate tiff files. The outline of the disc was selected using the magnetic lasso tool in Photoshop, and the number of pixels in the enclosed areas was measured. The number of green pixels within the disc was measured in ImageJ using the threshold tool at the default setting and then the analyze particles tool. The relative amount of transformed tissue in each disc was then calculated as: number of green pixels in disc/number of pixels in disc.

For each of these two calculations (cell counts for translocation, and disc area ratios for tissue overgrowth), values for each set of discs of a genotype of interest were compared to control, using Student's *t*-test when distributions were normal or Mann–Whitney u test when distributions were abnormal. For Fig. 2 and Fig. S2, two-tailed statistics were used. For the genetic screen, only samples with a mean greater than control were tested, and one-tailed statistics were used. We then calculated the false discovery rate (FDR)-adjusted *P*-values using the Benjamini-Hochberg method available in the Python package Statsmodels (https://doi.org/10.25080/Majora-92bf1922-011).

In the genetic screen, to account for variation in the baseline characteristics of the *ptc>Myc,p53^sh^* line over time (due to genetic drift) and slight variation in imaging settings from experiment to experiment, these statistical tests were first performed using only the controls from each individual experiment. However, as some experiments contained only a small number of samples for each genotype, and in order to calculate an FDR (above), we also performed the comparisons after aggregating the data for all experiments. Fly lines resulting in enhancement over control with *P*<0.05 in the individual experiment *and* FDR<0.1 in the aggregate were considered significant.

### Drugs

We used a library of drugs approved by the FDA to treat cancer, several drugs typically used to treat TNBC, as well as novel compounds developed in our laboratory, which showed anti-cancer activity in other fly thyroid and colorectal cancer models. Doses were approximations of the maximum tolerable dose, based on previous experience with other fly models, with lower doses of some drugs based on preliminary experiments on p53/Myc (Table S8). Drugs were dissolved in either water or DMSO, and then diluted 1:1000 by volume in the fly food. Water-soluble drugs were compared to a ‘water’ control for which no drug was added to the food. Drugs dissolved in DMSO were compared to food with 0.1% DMSO by volume.

### Drug experiments

We crossed *ptc-Gal4* to *w; Myc,p53^sh^* at 27°C, where the rate of eclosion on DMSO was 56%, lower than at 25°C. Flies were allowed to lay on four replicates of fresh drug food for each drug for 24 h. They were then flipped into another set of four replicates each and allowed to lay for 24 h, giving a total of eight replicates for each drug condition. The eclosion rate in percent was calculated as: 100×number of empty pupal cases/[number of uneclosed pupae+number of empty pupal cases].

The crosses used for experiments shown in Fig. 6 were performed similarly to those for the genetic screen. Virgin females of *hs-FLP/hs-hid; ptc-Gal4; Myc,p53^sh^/SM5(Gal80)-TM6B* were crossed with a control line comprising either genotype *w* or genes of interest identified in the screen. The eclosion rate in percent was calculated as described above only for non-tubby pupae. The relative pupariation rate in percent was also calculated: 100×[number of non-tubby pupae/number of tubby pupae]. Drug and food conditions were the same as above.

### Statistics

Fig. 1A includes both copy number and mutation data; copy number calculation is described above. For the purpose of clustering, mutation data were converted to numerals ranging from 0 to 2, with values <1 indicating likely loss of function and values >1 indicating likely gain of function, as follows: Missense Mutation=2.0, In-frame Insertion=1.9, In-frame Deletion=1.8, Nonstop Mutation=1.7, RNA Alteration=1.6, Splice Site mutation=0.3, Frameshift Insertion=0.2, Frameshift Deletion=0.1, Nonsense Mutation=0.0. Hierarchical clustering was then performed in Python 3 with the graphing package Seaborn (https://zenodo.org/record/883859#.Y3BVeuRBw2w) using the ‘clustermap’ function with the default Euclidean distance metric to generate Fig. 1A. To account for the effect of mixed data types on this clustering, we repeated this analysis using the original categorical designations for mutation type (i.e. ‘Missense Mutation’) and the same copy number data, and performed clustering using Gower distance (Gower, 1971) with the Python package Gower (https://zenodo.org/record/883859#.Y3BVeuRBw2w). This method also did not produce any discernible clusters. Code to reproduce both versions of the figure is available at https://github.com/jennifereldiaz/fly-tnbc.

Statistical tests were performed either in Python 3 with the statistics packages SciPy (http://www.scipy.org/) and Statsmodels (Seabold and Perktold, 2010) or Prism. In Fig. 6D, 2-way ANOVA and multiple *t*-tests (unpaired) functions in Prism 8 were used.

Survival analyses utilized the breast cancer portion of the TCGA Pan-Cancer clinical endpoints database (Liu et al., 2018) and were performed using the built-in log-rank test, Cox proportional hazard model and Kaplan–Meier curve function in the Python package Lifelines (CamDavidsonPilon/lifelines: v0.14.3, https://zenodo.org/record/1252342). Raw *P*-values from the log-rank test are shown in the Table S1 rather than false discovery rates, as this analysis is intended to complement the larger body of computational and functional studies on these genes rather than serve as a point of conclusion on its own.

## Supplementary Material

10.1242/dmm.050191_sup1 Supplementary information

Table S2. Results of MutSigCV analysis on TCGA breast cancer somatic mutation dataset.

Table S3. Computational data on all considered GISTIC 2.0 and ISAR genes.

Table S4. Fly lines and experimental data for tested genes in Group 4G and negative control genes.

Table S5. Summary of results for all CNA regions considered in this analysis. Regions in gray are not likely to be significant in TNBC, but may be relevant to other breast cancer subtypes; not all of these were studied to completion.

Table S6. Data from other databases for all considered GISTIC 2.0 and ISAR genes.

Table S7. Log-rank test hazard ratios (HR) and p-values for driver genes using the TCGA breast cancer dataset. PFI and OS are preferred by the authors of [39] and are the metrics shown in Figure 5. HR are reported as negative for deletions here, and this is corrected in Figure 5.

Table S8. Drugs used in the drug screen.

Table S9. Fly stocks and results for Group 1 genes Results symbols: +: p<0.05. ?: 0.05<p<0.4. -: p>0.4 or rescues phenotype. Each symbol represents one experiment.

Table S10. Fly stocks and results for Group 2 genes Results symbols: +: p<0.05. ?: 0.05<p<0.4. -: p>0.4 or rescues phenotype. Each symbol represents one experiment.

Table S11. Genetic screen results In the lethality column, only lines that were significant (p<0.05) in two independent tests are considered positive and reported in each numerator. In the validation column, ‘enhance’ refers to affecting an increase in the phenotype over *Myc,p53^sh^*. Lines were generally only tested for tissue overgrowth (called ‘growth’ in Supplemental Tables 9, 10) when negative for cell translocation; unless otherwise indicated, a test for tissue overgrowth implies a negative cell translocation test. ‘Passenger’ refers to genes lacking functional evidence for driver status in this study. ND = not done.

Table S12. Cell translocation assay results Stock numbers and statistical results corresponding to Figures 4a and S4a.

Table S13. Overgrowth assay results Stock numbers and statistical results corresponding to Figures 4b and S4b.

## References

[DMM050191C1] Akavia, U. D., Litvin, O., Kim, J., Sanchez-Garcia, F., Kotliar, D., Causton, H. C., Pochanard, P., Mozes, E., Garraway, L. A. and Pe'er, D. (2010). An integrated approach to uncover drivers of cancer. *Cell* 143, 1005-1017. 10.1016/j.cell.2010.11.013 21129771 PMC3013278

[DMM050191C2] Al-Lazikani, B., Banerji, U. and Workman, P. (2012). Combinatorial drug therapy for cancer in the post-genomic era. *Nat. Biotechnol.* 30, 679-692. 10.1038/nbt.2284 22781697

[DMM050191C3] Aran, D., Sirota, M. and Butte, A. J. (2017). Systematic pan-cancer analysis of tumour purity. *Nat. Commun.* 6, 8971. 10.1038/ncomms9971 PMC4671203 26634437

[DMM050191C4] Aure, M. R., Steinfeld, I., Baumbusch, L. O., Liestøl, K., Lipson, D., Nyberg, S., Naume, B., Sahlberg, K. K., Kristensen, V. N., Børresen-Dale, A.-L., et al. (2013). Identifying in-trans process associated genes in breast cancer by integrated analysis of copy number and expression data. *PLoS One* 8, e53014. 10.1371/journal.pone.0053014 23382830 PMC3559658

[DMM050191C5] Bangi, E., Murgia, C., Teague, A. G. S., Sansom, O. J. and Cagan, R. L. (2016). Functional exploration of colorectal cancer genomes using *Drosophila*. *Nat. Commun.* 7, 13615. 10.1038/ncomms13615 27897178 PMC5141297

[DMM050191C6] Bangi, E., Ang, C., Smibert, P., Uzilov, A. V., Teague, A. G., Antipin, Y., Chen, R., Hecht, C., Gruszczynski, N., Yon, W. J., et al. (2019). A personalized platform identifies trametinib plus zoledronate for a patient with KRAS-mutant metastatic colorectal cancer. *Sci. Adv.* 5, eaav6528. 10.1126/sciadv.aav6528 31131321 PMC6531007

[DMM050191C7] Bangi, E., Smibert, P., Uzilov, A. V., Teague, A. G., Gopinath, S., Antipin, Y., Chen, R., Hecht, C., Gruszczynski, N., Yon, W. J. et al. (2021). A *Drosophila* platform identifies a novel, personalized therapy for an adenoid cystic carcinoma patient. *iScience* 24, 102212. 10.1016/j.isci.2021.102212 33733072 PMC7940980

[DMM050191C8] Bauer, K. R., Brown, M., Cress, R. D., Parise, C. A. and Caggiano, V. (2007). Descriptive analysis of estrogen receptor (ER)-negative, progesterone receptor (PR)-negative, and HER2-negative invasive breast cancer, the so-called triple-negative phenotype. *Cancer* 109, 1721-1728. 10.1002/cncr.22618 17387718

[DMM050191C9] Braschi, B., Denny, P., Gray, K., Jones, T., Seal, R., Tweedie, S., Yates, B. and Bruford, E. (2019). Genenames.org: the HGNC and VGNC resources in 2019. *Nucleic Acids Res.* 47, D786-D792. 10.1093/nar/gky930 30304474 PMC6324057

[DMM050191C10] Cancer Genome Atlas Network (2012). Comprehensive molecular portraits of human breast tumours. *Nature* 490, 61-70. 10.1038/nature11412 23000897 PMC3465532

[DMM050191C11] Chandriani, S., Frengen, E., Cowling, V. H., Pendergrass, S. A., Perou, C. M., Whitfield, M. L. and Cole, M. D. (2009). A core MYC gene expression signature is prominent in basal-like breast cancer but only partially overlaps the core serum response. *PLoS One* 4, e6693. 10.1371/journal.pone.0006693 19690609 PMC2723908

[DMM050191C12] Ciriello, G., Miller, M. L., Aksoy, B. A., Senbabaoglu, Y., Schultz, N. and Sander, C. (2013). Emerging landscape of oncogenic signatures across human cancers. *Nat. Genet.* 45, 1127-1133. 10.1038/ng.2762 24071851 PMC4320046

[DMM050191C73] Ciriello, G., Gatza, M. L., Beck, A. H., Wilkerson, M. D., Rhie, S. K., Pastore, A., Zhang, H., McLellan, M., Yau, C., Kandoth, C., et al. (2015). Comprehensive Molecular Portraits of Invasive Lobular Breast Cancer. *Cell.* 163, 506-519. 10.1016/j.cell.2015.09.033 26451490 PMC4603750

[DMM050191C13] Curtis, C., Shah, S. P., Chin, S.-F., Turashvili, G., Rueda, O. M., Dunning, M. J., Speed, D., Lynch, A. G., Samarajiwa, S., Yuan, Y., et al. (2012). The genomic and transcriptomic architecture of 2,000 breast tumours reveals novel subgroups. *Nature* 486, 346-352. 10.1038/nature10983 22522925 PMC3440846

[DMM050191C14] Dar, A. C., Das, T. K., Shokat, K. M. and Cagan, R. L. (2012). Chemical genetic discovery of targets and anti-targets for cancer polypharmacology. *Nature* 486, 80-84. 10.1038/nature11127 22678283 PMC3703503

[DMM050191C15] de la Cova, C., Senoo-Matsuda, N., Ziosi, M., Wu, D. C., Bellosta, P., Quinzii, C. M. and Johnston, L. A. (2014). Supercompetitor status of *Drosophila* Myc cells requires p53 as a fitness sensor to reprogram metabolism and promote viability. *Cell Metab.* 19, 470-483. 10.1016/j.cmet.2014.01.012 24561262 PMC3970267

[DMM050191C16] Dent, R., Trudeau, M., Pritchard, K. I., Hanna, W. M., Kahn, H. K., Sawka, C. A., Lickley, L. A., Rawlinson, E., Sun, P. and Narod, S. A. (2007). Triple-negative breast cancer: clinical features and patterns of recurrence. *Clin. Cancer Res.* 13, 4429-4434. 10.1158/1078-0432.CCR-06-3045 17671126

[DMM050191C17] Flicek, P., Ridwan Amode, M., Barrell, D., Beal, K., Billis, K., Brent, S., Carvalho-Silva, D., Clapham, P., Coates, G., Fitzgerald, S., et al. (2014). Ensembl 2014. *Nucleic Acids Res.* 42, D749-D755. 10.1093/nar/gkt1196 24316576 PMC3964975

[DMM050191C18] Forbes, S. A., Beare, D., Gunasekaran, P., Leung, K., Bindal, N., Boutselakis, H., Ding, M., Bamford, S., Cole, C., Ward, S., et al. (2015). COSMIC: exploring the world's knowledge of somatic mutations in human cancer. *Nucleic Acids Res.* 43, D805-D811. 10.1093/nar/gku1075 25355519 PMC4383913

[DMM050191C19] Geisbrecht, E. R. and Montell, D. J. (2004). A role for *Drosophila* IAP1-mediated caspase inhibition in Rac-dependent cell migration. *Cell* 118, 111-125. 10.1016/j.cell.2004.06.020 15242648

[DMM050191C20] Gladstone, M., Frederick, B., Zheng, D., Edwards, A., Yoon, P., Stickel, S., DeLaney, T., Chan, D. C., Raben, D. and Su, T. T. (2012). A translation inhibitor identified in a *Drosophila* screen enhances the effect of ionizing radiation and taxol in mammalian models of cancer. *Dis. Model. Mech.* 5, 342-350. 22344740 10.1242/dmm.008722 PMC3339828

[DMM050191C21] Gorelick-Ashkenazi, A., Weiss, R., Sapozhnikov, L., Florentin, A., Tarayrah-Ibraheim, L., Dweik, D., Yacobi-Sharon, K. and Arama, E. (2018). Caspases maintain tissue integrity by an apoptosis-independent inhibition of cell migration and invasion. *Nat. Commun.* 9, 2806. 10.1038/s41467-018-05204-6 30022065 PMC6052023

[DMM050191C22] Gower, J. C. (1971). A general coefficient of similarity and some of its properties. *Biometrics* 27, 857-871. 10.2307/2528823

[DMM050191C23] Griffith, M., Spies, N. C., Krysiak, K., McMichael, J. F., Coffman, A. C., Danos, A. M., Ainscough, B. J., Ramirez, C. A., Rieke, D. T., Kujan, L., et al. (2017). CIViC is a community knowledgebase for expert crowdsourcing the clinical interpretation of variants in cancer. *Nat. Genet.* 49, 170-174. 10.1038/ng.3774 28138153 PMC5367263

[DMM050191C24] Harbeck, N. and Gnant, M. (2017). Breast cancer. *Lancet* 389, 1134-1150. 10.1016/S0140-6736(16)31891-8 27865536

[DMM050191C25] Hirabayashi, S., Baranski, T. J. and Cagan, R. L. (2013). Transformed *Drosophila* cells evade diet-mediated insulin resistance through wingless signaling. *Cell* 154, 664-675. 10.1016/j.cell.2013.06.030 23911328 PMC3800019

[DMM050191C26] Hu, Y., Flockhart, I., Vinayagam, A., Bergwitz, C., Berger, B., Perrimon, N. and Mohr, S. E. (2011). An integrative approach to ortholog prediction for disease-focused and other functional studies. *BMC Bioinformatics* 12, 357. 10.1186/1471-2105-12-357 21880147 PMC3179972

[DMM050191C27] Hyman, D. M., Taylor, B. S. and Baselga, J. (2017). Implementing genome-driven oncology. *Cell* 168, 584-599. 10.1016/j.cell.2016.12.015 28187282 PMC5463457

[DMM050191C28] Jiang, Y.-Z., Ma, D., Suo, C., Shi, J., Xue, M., Hu, X., Xiao, Y., Yu, K.-D., Liu, Y.-R., Yu, Y., et al. (2019). Genomic and transcriptomic landscape of triple-negative breast cancers: subtypes and treatment strategies. *Cancer Cell* 35, 428-440.e5. 10.1016/j.ccell.2019.02.001 30853353

[DMM050191C29] Johnson, M., Zaretskaya, I., Raytselis, Y., Merezhuk, Y., McGinnis, S. and Madden, T. L. (2008). NCBI BLAST: a better web interface. *Nucleic Acids Res.* 36, W5-W9. 10.1093/nar/gkn201 18440982 PMC2447716

[DMM050191C31] Kessler, J. D., Kahle, K. T., Sun, T., Meerbrey, K. L., Schlabach, M. R., Schmitt, E. M., Skinner, S. O., Xu, Q., Li, M. Z., Hartman, Z. C., et al. (2012). A SUMOylation-dependent transcriptional subprogram is required for Myc-driven tumorigenesis. *Science* 335, 348-353. 10.1126/science.1212728 22157079 PMC4059214

[DMM050191C32] Koedoot, E., Fokkelman, M., Rogkoti, V.-M., Smid, M., van de Sandt, I., de Bont, H., Pont, C., Klip, J. E., Wink, S., Timmermans, M. A., et al. (2019). Uncovering the signaling landscape controlling breast cancer cell migration identifies novel metastasis driver genes. *Nat. Commun.* 10, 2983. 10.1038/s41467-019-11020-3 31278301 PMC6611796

[DMM050191C33] Lawrence, M. S., Stojanov, P., Polak, P., Kryukov, G. V., Cibulskis, K., Sivachenko, A., Carter, S. L., Stewart, C., Mermel, C. H., Roberts, S. A., et al. (2013). Mutational heterogeneity in cancer and the search for new cancer-associated genes. *Nature* 499, 214-218. 10.1038/nature12213 23770567 PMC3919509

[DMM050191C34] Lei, K.-F., Wang, Y.-F., Zhu, X.-Q., Lu, P.-C., Sun, B.-S., Jia, H.-L., Ren, N., Ye, Q.-H., Sun, H.-C., Wang, L., et al. (2007). Identification of MSRA gene on chromosome 8p as a candidate metastasis suppressor for human hepatitis B virus-positive hepatocellular carcinoma. *BMC Cancer* 7, 172. 10.1186/1471-2407-7-172 17784942 PMC2000900

[DMM050191C35] Levine, B. D. and Cagan, R. L. (2016). *Drosophila* lung cancer models identify trametinib plus statin as candidate therapeutic. *Cell Rep.* 14, 1477-1487. 10.1016/j.celrep.2015.12.105 26832408 PMC4904304

[DMM050191C36] Levinson, S. and Cagan, R. L. (2016). *Drosophila* cancer models identify functional differences between ret fusions. *Cell Rep.* 16, 3052-3061. 10.1016/j.celrep.2016.08.019 27626672 PMC5858711

[DMM050191C37] Li, X., Chen, X., Hu, G., Liu, Y., Zhang, Z., Wang, P., Zhou, Y., Yi, X., Zhang, J., Zhu, Y. et al. (2014). Combined analysis with copy number variation identifies risk loci in lung cancer. *BioMed Res. Int.* 2014, 1-9. 10.1155/2014/469103 PMC4100386 25093167

[DMM050191C74] Li, B. and Dewey, C.N. (2011). RSEM: accurate transcript quantification from RNA-Seq data with or without a reference genome. BMC Bioinformatics 12, 323. 10.1186/1471-2105-12-323 21816040 PMC3163565

[DMM050191C38] Liu, J., Lichtenberg, T., Hoadley, K. A., Poisson, L. M., Lazar, A. J., Cherniack, A. D., Kovatich, A. J., Benz, C. C., Levine, D. A., Lee, A. V. et al. (2018). An integrated TCGA pan-cancer clinical data resource to drive high-quality survival outcome analytics. *Cell* 173, 400-416.e11. 10.1016/j.cell.2018.02.052 29625055 PMC6066282

[DMM050191C39] Lobry, C., Oh, P., Mansour, M. R., Look, A. T. and Aifantis, I. (2014). Notch signaling: switching an oncogene to a tumor suppressor. *Blood* 123, 2451-2459. 10.1182/blood-2013-08-355818 24608975 PMC3990910

[DMM050191C40] Luca, A. D., De Luca, A., Sanna, F., Sallese, M., Ruggiero, C., Grossi, M., Sacchetta, P., Rossi, C., De Laurenzi, V., Di Ilio, C. et al. (2010). Methionine sulfoxide reductase A down-regulation in human breast cancer cells results in a more aggressive phenotype. *Proc. Natl Acad. Sci. USA* 107, 18628-18633. 10.1073/pnas.1010171107 20937881 PMC2972941

[DMM050191C41] Marcotte, R., Sayad, A., Brown, K. R., Sanchez-Garcia, F., Reimand, J., Haider, M., Virtanen, C., Bradner, J. E., Bader, G. D., Mills, G. B. et al. (2016). Functional genomic landscape of human breast cancer drivers, vulnerabilities, and resistance. *Cell* 164, 293-309. 10.1016/j.cell.2015.11.062 26771497 PMC4724865

[DMM050191C42] Markstein, M., Dettorre, S., Cho, J., Neumüller, R. A., Craig-Müller, S. and Perrimon, N. (2014). Systematic screen of chemotherapeutics in *Drosophila* stem cell tumors. *Proc. Natl. Acad. Sci. USA* 111, 4530-4535. 10.1073/pnas.1401160111 24616500 PMC3970492

[DMM050191C43] Marra, A., Viale, G. and Curigliano, G. (2019). Recent advances in triple negative breast cancer: the immunotherapy era. *BMC Med.* 17, 90. 10.1186/s12916-019-1326-5 31068190 PMC6507064

[DMM050191C44] Mermel, C. H., Schumacher, S. E., Hill, B., Meyerson, M. L., Beroukhim, R. and Getz, G. (2011). GISTIC2.0 facilitates sensitive and confident localization of the targets of focal somatic copy-number alteration in human cancers. *Genome Biol.* 12, R41. 10.1186/gb-2011-12-4-r41 21527027 PMC3218867

[DMM050191C45] Miao, K., Lei, J. H., Valecha, M. V., Zhang, A., Xu, J., Wang, L., Lyu, X., Chen, S., Miao, Z., Zhang, X. et al. (2020). NOTCH1 activation compensates BRCA1 deficiency and promotes triple-negative breast cancer formation. *Nat. Commun.* 11, 3256. 10.1038/s41467-020-16936-9 32591500 PMC7320176

[DMM050191C47] Mount, D. W. (2007). Using the basic local alignment search tool (BLAST). *CSH Protoc.* 10.1101/pdb.top17 21357135

[DMM050191C48] Murali, T., Pacifico, S., Yu, J., Guest, S., Roberts, G. G. 3rd and Finley, R. L. Jr. (2011). DroID 2011: a comprehensive, integrated resource for protein, transcription factor, RNA and gene interactions for *Drosophila*. *Nucleic Acids Res.* 39, D736-D743. 10.1093/nar/gkq1092 21036869 PMC3013689

[DMM050191C49] Muss, H. B., Berry, D. A., Cirrincione, C. T., Theodoulou, M., Mauer, A. M., Kornblith, A. B., Partridge, A. H., Dressler, L. G., Cohen, H. J., Becker, H. P. et al. (2009). Adjuvant chemotherapy in older women with early-stage breast cancer. *N. Engl. J. Med.* 360, 2055-2065. 10.1056/NEJMoa0810266 19439741 PMC3082436

[DMM050191C50] Nagahashi, M., Ling, Y., Hayashida, T., Kitagawa, Y., Futamura, M., Yoshida, K., Kuwayama, T., Nakamura, S., Toshikawa, C., Yamauchi, H. et al. (2018). Actionable gene alterations in an Asian population with triple-negative breast cancer. *JCO Precis. Oncol.* 2, PO.17.00211. 10.1200/po.17.00211 32529167 PMC7288901

[DMM050191C51] Parsons, D. W., Jones, S., Zhang, X., Lin, J. C.-H., Leary, R. J., Angenendt, P., Mankoo, P., Carter, H., Siu, I.-M., Gallia, G. L. et al. (2008). An integrated genomic analysis of human glioblastoma multiforme. *Science* 321, 1807-1812. 10.1126/science.1164382 18772396 PMC2820389

[DMM050191C52] Patel, N., Weekes, D., Drosopoulos, K., Gazinska, P., Noel, E., Rashid, M., Mirza, H., Quist, J., Brasó-Maristany, F., Mathew, S. et al. (2018). Integrated genomics and functional validation identifies malignant cell specific dependencies in triple negative breast cancer. *Nat. Commun.* 9, 1044. 10.1038/s41467-018-03283-z 29535384 PMC5849766

[DMM050191C53] Pfeifer, D., Pantic, M., Skatulla, I., Rawluk, J., Kreutz, C., Martens, U. M., Fisch, P., Timmer, J. and Veelken, H. (2006). Genome-wide analysis of DNA copy number changes and LOH in CLL using high-density SNP arrays. *Blood* 109, 1202-1210. 10.1182/blood-2006-07-034256 17053054

[DMM050191C54] Pina, C. and Pignoni, F. (2012). Tubby-RFP balancers for developmental analysis: FM7c 2xTb-RFP, CyO 2xTb-RFP, and TM3 2xTb-RFP. *Genesis* 50, 119-123. 10.1002/dvg.20801 21913310 PMC3931234

[DMM050191C55] Read, R. D., Goodfellow, P. J., Mardis, E. R., Novak, N., Armstrong, J. R. and Cagan, R. L. (2005). A *Drosophila* model of multiple endocrine neoplasia type 2. *Genetics* 171, 1057-1081. 10.1534/genetics.104.038018 15965261 PMC1456812

[DMM050191C56] Rudrapatna, V. A., Bangi, E. and Cagan, R. L. (2013). Caspase signalling in the absence of apoptosis drives Jnk-dependent invasion. *EMBO Rep.* 14, 172-177. 10.1038/embor.2012.217 23306653 PMC3596137

[DMM050191C57] Sanchez-Garcia, F., Villagrasa, P., Matsui, J., Kotliar, D., Castro, V., Akavia, U.-D., Chen, B.-J., Saucedo-Cuevas, L., Barrueco, R. R., Llobet-Navas, D. et al. (2014). Integration of genomic data enables selective discovery of breast cancer drivers. *Cell* 159, 1461-1475. 10.1016/j.cell.2014.10.048 25433701 PMC4258423

[DMM050191C58] Schroeder, M. P., Rubio-Perez, C., Tamborero, D., Gonzalez-Perez, A. and Lopez-Bigas, N. (2014). OncodriveROLE classifies cancer driver genes in loss of function and activating mode of action. *Bioinformatics* 30, i549-i555. 10.1093/bioinformatics/btu467 25161246 PMC4147920

[DMM050191C61] Shen, L., Shi, Q. and Wang, W. (2018). Double agents: genes with both oncogenic and tumor-suppressor functions. *Oncogenesis* 7, 25. 10.1038/s41389-018-0034-x 29540752 PMC5852963

[DMM050191C46] Siegel, R. L., Miller, K. D. and Jemal, A. (2018). Cancer statistics, 2018. *CA Cancer J. Clin.* 68, 7-30. 10.3322/caac.21442 29313949

[DMM050191C62] Sonoshita, M., Scopton, A. P., Ung, P. M. U., Murray, M. A., Silber, L., Maldonado, A. Y., Real, A., Schlessinger, A., Cagan, R. L. and Dar, A. C. (2018). A whole-animal platform to advance a clinical kinase inhibitor into new disease space. *Nat. Chem. Biol.* 14, 291-298. 10.1038/nchembio.2556 29355849 PMC5931369

[DMM050191C63] Taylor, B. S., Barretina, J., Socci, N. D., DeCarolis, P., Ladanyi, M., Meyerson, M., Singer, S. and Sander, C. (2008). Functional copy-number alterations in cancer. *PLoS One* 3, e3179. 10.1371/journal.pone.0003179 18784837 PMC2527508

[DMM050191C64] Vidal, M., Wells, S., Ryan, A. and Cagan, R. (2005). ZD6474 suppresses oncogenic RET isoforms in a *Drosophila* model for type 2 multiple endocrine neoplasia syndromes and papillary thyroid carcinoma. *Cancer Res.* 65, 3538-3541. 10.1158/0008-5472.CAN-04-4561 15867345

[DMM050191C65] Vidal, M., Larson, D. E. and Cagan, R. L. (2006). Csk-deficient boundary cells are eliminated from normal *Drosophila* epithelia by exclusion, migration, and apoptosis. *Dev. Cell* 10, 33-44. 10.1016/j.devcel.2005.11.007 16399076

[DMM050191C66] Vogelstein, B., Papadopoulos, N., Velculescu, V. E., Zhou, S., Diaz, L. A., Jr. and Kinzler, K. W. (2013). Cancer genome landscapes. *Science* 339, 1546-1558. 10.1126/science.1235122 23539594 PMC3749880

[DMM050191C67] Walerych, D., Napoli, M., Collavin, L. and Del Sal, G. (2012). The rebel angel: mutant p53 as the driving oncogene in breast cancer. *Carcinogenesis* 33, 2007-2017. 10.1093/carcin/bgs232 22822097 PMC3483014

[DMM050191C69] Waterhouse, R. M., Tegenfeldt, F., Li, J., Zdobnov, E. M. and Kriventseva, E. V. (2013). OrthoDB: a hierarchical catalog of animal, fungal and bacterial orthologs. *Nucleic Acids Res.* 41, D358-D365. 10.1093/nar/gks1116 23180791 PMC3531149

[DMM050191C70] Willoughby, L. F., Schlosser, T., Manning, S. A., Parisot, J. P., Street, I. P., Richardson, H. E., Humbert, P. O. and Brumby, A. M. (2013). An in vivo large-scale chemical screening platform using *Drosophila* for anti-cancer drug discovery. *Dis. Model. Mech* 6, 521-529. 22996645 10.1242/dmm.009985 PMC3597034

[DMM050191C71] Wu, J., Klein, T. J. and Mlodzik, M. (2004). Subcellular localization of frizzled receptors, mediated by their cytoplasmic tails, regulates signaling pathway specificity. *PLoS Biol.* 2, E158. 10.1371/journal.pbio.0020158 15252441 PMC449784

