# Supplementary material for: Functional exploration of copy number alterations in a Drosophila model of triple-negative breast cancer
Source: Dis Model Mech. 2024 Jul 3;17(7):dmm050191. doi: 10.1242/dmm.050191 (PMC11247506; doi:10.1242/dmm.050191)
Supplement: Supplementary information [file dmm-17-050191-s1.pdf]

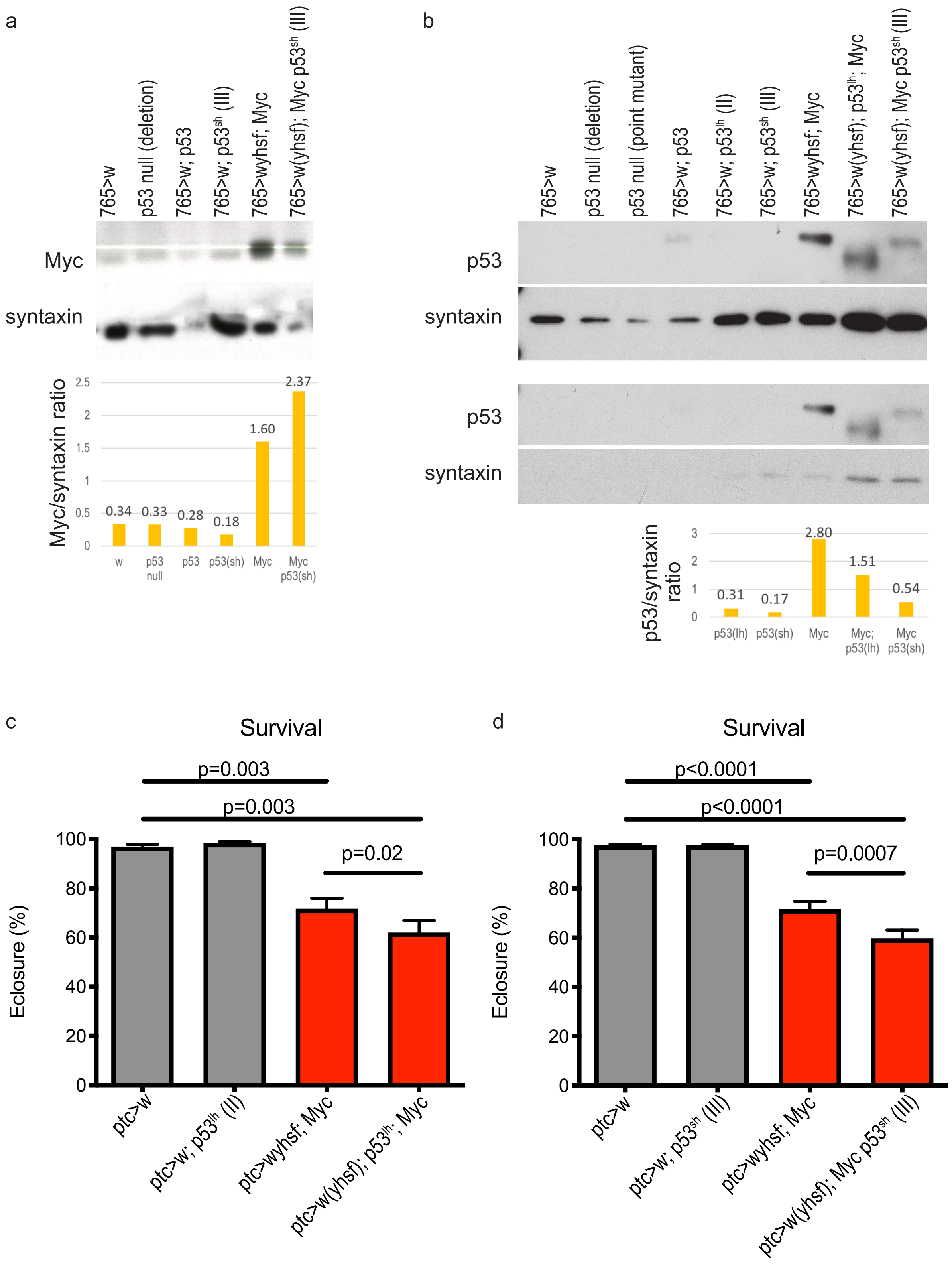

Disease Models & Mechanisms • Supplementary information

Fig. S1. Myc and p53-RNAi synergize to impact survival in *Drosophila*

a) Western blot of Myc in fly wing discs with quantification below. b) Western blot of p53 in fly wing discs. Due to loading variability, two sets of exposures are shown for clarity. The lower set of exposures were used for quantification, below. See Materials and Methods section for discussion of the faster migrating P53 band. c) Survival of Myc-expressing flies when combined with $p53^{lh}$. d) Survival of *Myc*-expressing flies when combined with $p53^{sh}$. In c-d, Kruskal-Wallis test p<0.0001 and N=11. P-values reflect Wilcoxon tests. *y* and *hs-flip* elements were present on the X chromosome where denoted. Related to Figure 1.

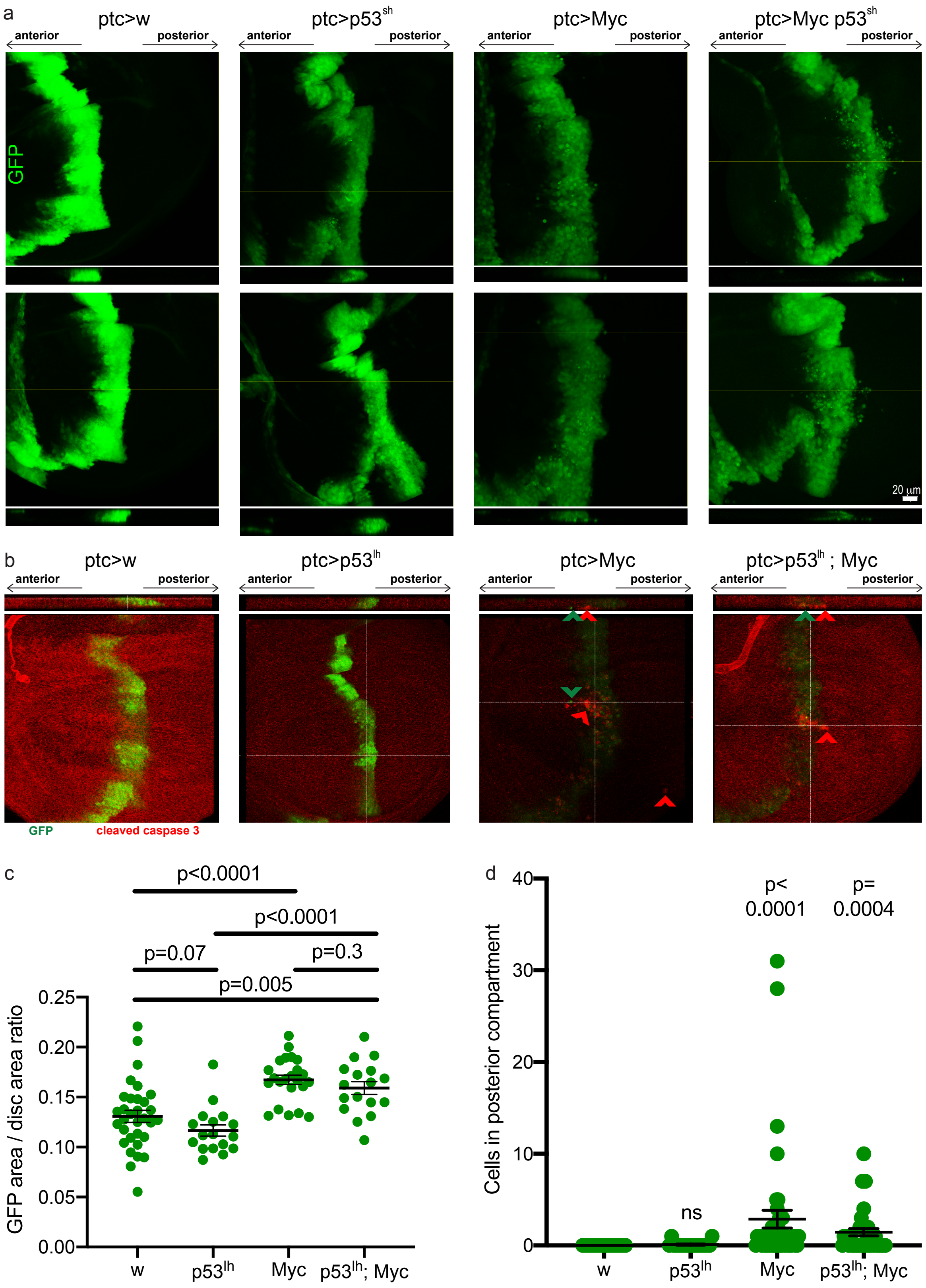
a
ptc>w
ptc>p53sh
ptc>Myc
ptc>Myc p53sh
anterior
posterior
GFP
20 µm
b
ptc>w
ptc>p53lh
ptc>Myc
ptc>p53lh ; Myc
anterior
posterior
GFP
cleaved caspase 3
c
p<0.0001
p<0.0001
p=0.07
p=0.3
p=0.005
GFP area / disc area ratio
0.25
0.20
0.15
0.10
0.05
0.00
w
p53lh
Myc
p53lh; Myc
d
Cells in posterior compartment
40
30
20
10
0
p<
0.0001
p=
0.0004
ns
w
p53lh
Myc
p53lh; Myc

Fig. S2. Overexpression of Myc induced tissue expansion and cell translocation in *Drosophila* wing discs

Maximum projections (bottom) and z-stacks (top) of confocal stacks of the lower half of wing discs (a) showing tissue overgrowth and cell translocation for the same genotypes in Figure 2, and (b) wing discs stained with a cleaved-caspase antibody (red) for the same genotypes shown in Supplemental Figure 1c. Arrowheads mark delaminating or migrating cells; brightness and contrast were uniformly increased to improve visualization of staining. In (b), note $p53^{lh}$ was used rather than $p53^{sh}$ (Figure 2). Magnification: 40X. Anterior at left, posterior at right, apical at top, basal at bottom. c) Quantification of transgenic tissue overgrowth produced by combinations of *Myc* and $p53^{lh}$ driven by *ptc-Gal4*. Kruskal-Wallis test: p<0.0001. P-values reflect student's t tests. d) Quantification of cell translocation in transgenic tissue produced by combinations of *Myc* and $p53^{lh}$ driven by *ptc-Gal4*. Kruskal-Wallis test: p<0.0001 P-values reflect Mann-Whitney tests compared to *w*. No significant difference was seen between *Myc* and $p53^{lh}$*; Myc*. Related to Figure 2.

Disease Models & Mechanisms • Supplementary information

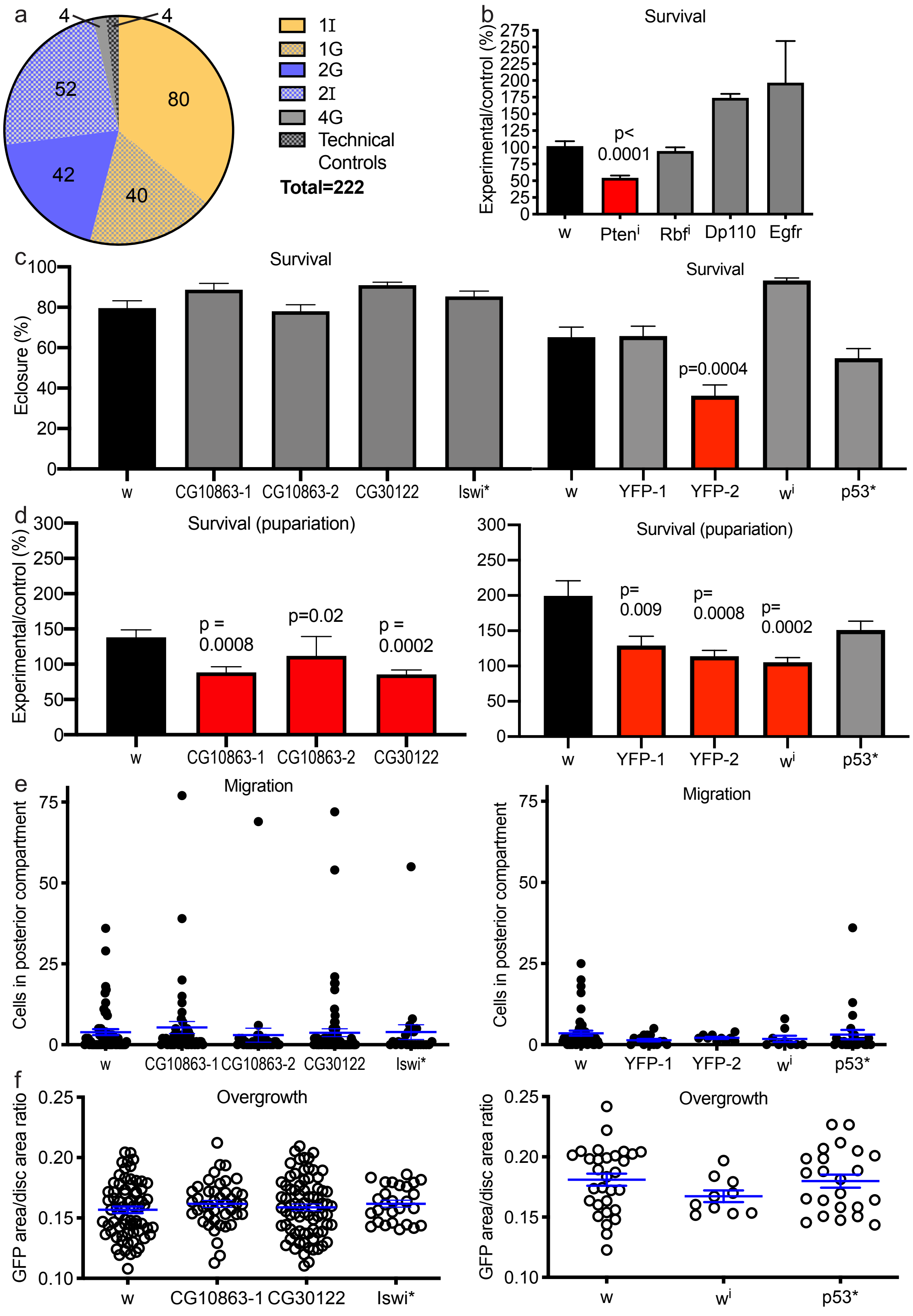

Disease Models & Mechanisms • Supplementary information

Fig. S3. Characterization of positive and negative controls in three *Drosophila* assays

a) Distribution of fly lines assessed in the screen among 6 computationally defined groups. b) Survival of flies to pupariation for positive controls (n=4 for EGFR, n=8 otherwise). c-d) Survival of flies to eclosure (c) and pupariation (d) for low priority genes (left panels: n=8 for CG10863-2 and Iswi*, n=24 for w, n=16 otherwise) and negative controls (right panels: n=8 for all). Pupariation could not be assessed in Iswi* because of a balancer. CG10863-1 and -2 are two lines for the same gene. The pupariation calculation is normalized by counts of internal control pupae and may be more sensitive to noise. e) Quantification of cell translocation in Group 4G (left) and control (right) lines. f) Quantification of overgrowth of transgenic tissue in Group 4G (left) and control (right) lines. Overgrowth could not be assessed in CG10863-2 because of a GFP tag, nor the YFP lines. All genotypes shown are in a background of $Myc,p53^{sh}$. p-values reflect a student's t test where data are normally distributed, or a Mann-Whitney test otherwise, compared to *w*. Blue error bars indicate a non-significant difference. Related to Figure 3. See also Supplemental Table 4.

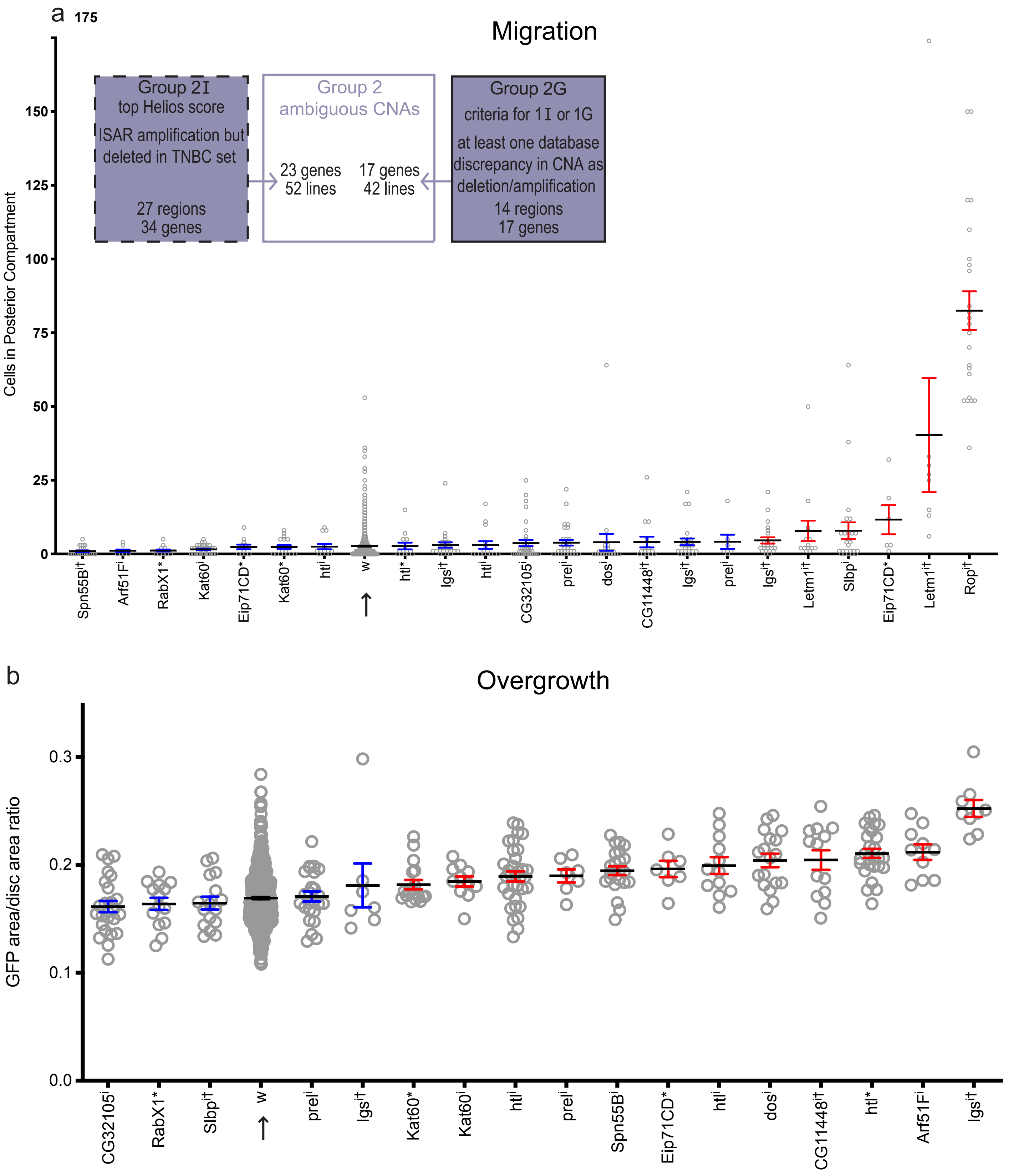

Disease Models & Mechanisms • Supplementary information

Fig. S4. Some driver genes that appear in ambiguous CNAs produce tissue phenotypes in the background of *Myc* and *p53*^{sh}

a) Quantification of cell translocation for genes from Group 2. Genes marked in red cause significant increase compared to *w* (arrow), measured as $p < 0.05$ in the original experiment and false discovery rate (fdr) < 0.1 in this aggregate analysis. b) Quantification of transgenic tissue overgrowth for genes from ambiguous deletions. Genes marked in red cause significant increase compared to *w* (arrow), measured as $p < 0.05$ in the original experiment and fdr < 0.1 in this aggregate analysis. Because of variation from one experiment to another, some genes that appear significant in this figure were not significant in their respective experiments. i indicates RNAi against the listed gene; * indicates a heterozygous null allele. † indicates Group 2G; the rest are Group 2I (see Methods). Related to Figure 4.

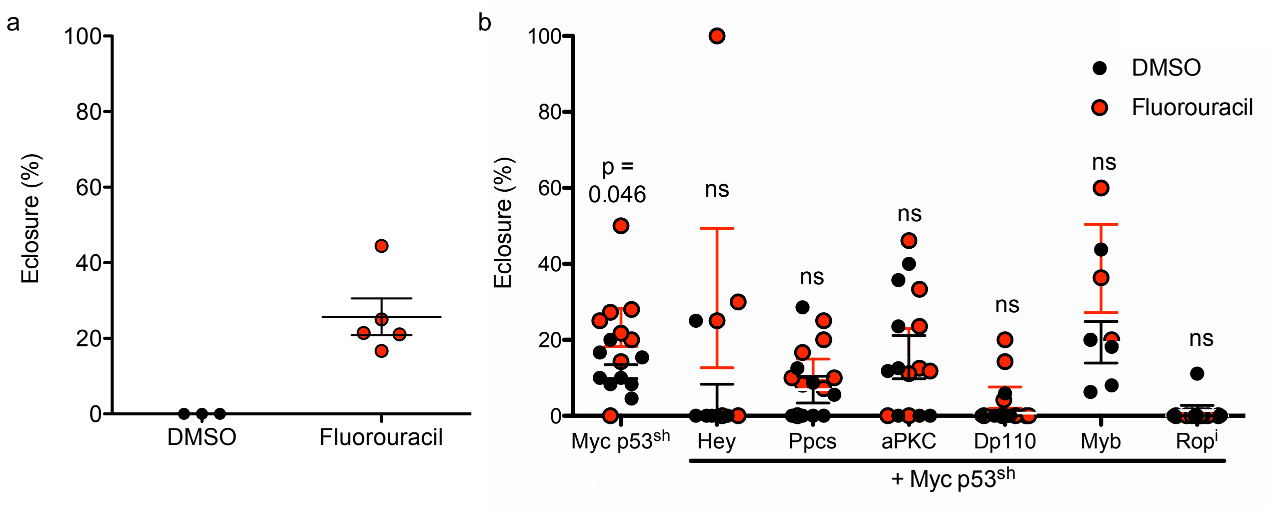

Fig. S5. Genetic modifiers abrogate the survival response of *p53^{sh} Myc* to fluorouracil at 29 °C

Fluorouracil was tested on the ptc>Myc,p53^{sh} line at 29 °C (a) and and ptc>Myc,p53^{sh} plus six selected driver genes at 29 °C (b). In each case tested, addition of an additional driver led to loss of fluorouracil-mediated rescue. d) Fluorouracil was tested at two doses on *ptc>w*, *ptc>Myc,p53^{sh}*, *ptc>Myc,p53^{sh},Myb*, and *ptc>Myc,p53^{sh},Dp110*, and transgenic tissue overgrowth was quantified as in Figure 4b. Two-way ANOVA results (b) were genotype: $p<0.0001$, drug: $p=0.001$, interaction: ns. Displayed p-values reflect t tests (see Methods). The final N for each condition is shown on each bar. Related to Figure 6.

Disease Models & Mechanisms • Supplementary information

Gene	Chr	Region	CNV type	Percent Altered	t-test Result	CN-RNA relationship	CNV type consistent with dataset	Fly gene	Lethality	Tissue	Other databases	MutSigCV q-value	Survival analysis HR (p value)
AKIRIN1	1	ISAR 2	amp	23.8	*	+	yes	akirin	1/1 overexpression line	1/1 line enhanced cell migration	RPPA essential (7 clusters)		PFI 2.02 (0.01)
HEYL			amp	23.8	ns	-	yes	Hey	1/1 overexpression lines	1/1 line causes tumor	RPPA essential (7 clusters)		PFI 1.85 (0.02)
PPCS		ISAR 3	amp	23.8	*	+	yes	Ppcs	2/3 overexpression lines	1/1 line enhanced cell migration			PFI 1.91 (0.01)
TM2D1		ISAR 4	amp	19.0	ns	+	yes	amx	1/1 overexpression line and 1/1 duplication line	1/1 line enhanced tissue overgrowth			
INADL			amp	18.1	ns	+	yes	dlt	1/2 overexpression lines	1/1 line enhanced tissue overgrowth			
ADAM15		ISAR 7, basal amp 1q22	amp	69.5	*	+	yes	Meltrin	1/3 overexpression lines	1/2 lines enhanced migration, 1/1 line enhanced tissue overgrowth			
RBM34		ISAR 10, total amp 1q44	amp	61.0	*	+	yes	Pabp2	0/1 overexpression line	1/1 overexpression line enhanced tissue overgrowth, 1/1 HA-tagged line (and 0/1 nontagged line) enhanced cell migration			
GRHL1	2	ISAR 11, basal del 2p25.3, total del 2p25.1	amp	24.8	*	+	yes	grh	1/1 overexpression line	1/1 line enhanced cell migration and tissue overgrowth			OS 1.58 (0.09)
KLF11			amp	24.8	*	+	yes	cbt	2/2 overexpression lines	1/1 line enhanced cell migration			
PRKCI	3	ISAR 15, basal amp 3q26.32	amp	50.5	*	+	yes	aPKC	3/4 overexpression lines	3/4 lines enhanced cell migration, 1/1 line enhanced tissue overgrowth			
TBL1XR1		basal amp 3q26.32, total amp 3q26.32	amp	47.6	*	+	yes	ebi	2/3 overexpression lines	2/3 lines enhanced cell migration		2.09E-03	
PIK3CA		ISAR 16, basal amp 3q26.32, total amp 3q26.32	amp	43.8	*	+	yes	Pi3K92E	3/3 overexpression lines	2/2 lines enhanced cell migration, 2/2 lines enhanced tissue overgrowth	Cosmic, TCGA pan-cancer, Parsons et al, CiVIC	7.76E-14	
SLBP	4	ISAR 17, basal del 4p16.3	del	48.6	*	+	yes	Slbp	1/1 RNAi line	1/1 line enhanced cell migration	Decreased expression essential, Heterozygous copy loss essential		
LETM1			del	48.6	*	+	yes	Letm1	1/2 RNAi lines, 0/1 mutated line	2/2 lines enhanced cell migration			
GAB1		ISAR 20	del	36.2	*	+	no	dos	1/1 RNAi line, 0/2 disrupted lines	1/1 line enhanced tissue overgrowth			
TRIO	5	ISAR 22	amp	32.4	*	+	yes	trio	2/2 partial cDNA overexpression lines, 0/2 full cDNA overexpression ine	1/1 full cDNA overexpression line enhanced cell migration	Basal essential		
MIER3		basal del 5q11.2, total del 5q11.2	del	70.5	*	+	yes	CG1620	1/1 line (unconfirmed)	1/1 line enhanced cell migration			
FGFR4		ISAR 24	del	44.8	ns	+	no	htl	see FGFR1				
DEK	6	basal amp 6p22.3, total amp 6p23	amp	40.0	*	+	yes	Dek	1/1 overexpression line	1/1 line enhanced tissue overgrowth	Cosmic		
E2F3		basal amp 6p22.3	amp	41.9	*	+	yes	E2f	4/5 overexpression lines	1/3 lines enhanced cell migration	RPPA essential (7 clusters), Basal essential, Increased expression essential		
SOX4			amp	43.8	*	+	yes	Sox14	1/2 overexpression lines	1/1 lines enhanced tissue overgrowth	Myc-SL		
FOXP4		ISAR 25, basal amp 6p21.1	amp	42.9	*	+	yes	FoxP	1/1 overexpression line	1/1 line enhanced tissue overgrowth	Basal essential		
PTP4A1		ISAR 27, total del 6q14.3	amp	26.7	ns	+	yes	PRL-1	1/2 overexpression lines	1/1 line enhanced cell migration			
C6orf203		ISAR 28, basal amp 6q21	amp	31.4	*	+	yes	CG4884	1/1 overexpression line	1/1 line enhanced tissue overgrowth			OS 2.17 (0.0004)
MYB*		ISAR 29, basal amp 6q23.3, basal del 6q25.3	amp	28.6	ns	-	yes	Myb	1/2 overexpression lines, 1/1 RNAi line, 0/2 mutated lines	1/1 RNAi line enhanced migration, 1/1 overexpression line enhanced tissue overgrowth	Cosmic	0.44991	OS 1.66 (0.04)
EGFR	7	ISAR 34, basal amp 7p11.2, total amp 7p11.2	amp	22.9	ns	+	yes	Egfr	2/5 overexpression lines	2/2 lines enhanced cell migration, 1/1 line enh tissue overgrowth	Cosmic, TCGA pan-cancer, Parsons et al, CiVIC, Neve sub-type essential (basal A &I B, HER2 essential, luminal), RPPA essential (7 clusters), Basal essential, Increased expression essential		
AUTS2		ISAR 35	amp	16.2	*	+	yes	tay	2/2 overexpression lines	1/1 line enhanced cell migration	Basal essential		
UBE3C		ISAR 37	amp	21.0	*	+	yes	CG3356	2/2 overexpression lines	1/2 lines enhanced cell migration, 1/1 line enhanced tissue overgrowth			
DNAJB6			amp	22.9	*	+	yes	mrj	1/2 overexpression lines	1/1 line enhanced tissue overgrowth			
MSRA	8	ISAR 38	del	56.2	*	+	no	Eip71CD	2/3 disrupted lines	1/2 disrupted lines enhanced cell migration			OS 1.51 (0.02)
LETM2		basal amp 8p11.23, basal del 8p22	del	29.5	*	+	yes	Letm1	see LETM1		Basal essential, Neve subtypes essential (basal A & B, HER2, luminal)		
FGFR1			del	29.5	*	+	yes	htl	1/2 RNAi lines, 1/1 amorphic line	1/1 RNAi line and 1/1 amorphic line enhanced tissue overgrowth	Cosmic, TCGA pan-cancer, CiVIC, Aure et al, Basal essential, Increase expression essential		
ANKRD46		ISAR 41	amp	72.4	*	+	yes	CG10809	2/2 overexpression lines	2/2 lines enhanced tissue overgrowth, 0/3 lines enhanced cell migration	Increased expression essential		OS 1.40 (0.05)
GRHL2 †			amp	69.5	*	+	yes	grh	see GHRL1			0.45446	OS 1.39 (0.06)
TRPS1		ISAR 42	amp	61.9	*	+	yes	srp	3/4 overexpression lines	1/2 lines enhanced migration	Myc-SL		OS 1.47 (0.03)
MYC		ISAR 43, basal amp 8q24.21, total amp 8q24.21	amp	76.2	*	+	yes	dm	see figure 1	see figure 2	Cosmic, Parsons et al, RPPA essential (7 clusters)		OS 1.45 (0.04)
STXBP1	9	ISAR 45, basal del 9q34.3	del	42.9	*	+	yes	Rop	1/1 RNAi line	1/1 line enhanced cell migration			
PTEN	10	basal del 10q23.31, total del 10q23.31	del	34.3	*	+	yes	Pten	3/4 RNAi lines, 0/1 disrupted line	1/1 RNAi line enhanced cell migration and tissue overgrowth, 0/1 disrupted line	Cosmic, TCGA pan-cancer, Parsons et al, CiVIC, Myc-SL	0	
BCL9L	11	ISAR 54, basal del 11q25	del	38.1	*	+	yes	lgs	3/3 RNAi lines	1/2 lines enhanced cell migration, 1/1 lines enhanced tissue overgrowth			
ETV6	12	basal amp 12p13.2, total del 12p13.2	amp	22.9	*	+	yes	aop	2/2 overexpression lines	1/1 line enhanced tissue overgrowth	Cosmic, TCGA pan-cancer		
RILPL2		ISAR 59, basal del 12q24.31, total del 12q24.33	del	31.4	*	+	yes	CG11448	2/2 RNAi lines	1/1 line enhanced tissue overgrowth			
KATNAL1	13	ISAR 61, total amp 13q12.3	del	42.9	*	+	no	Kat60	1/1 RNAi line, 1/1 disrupted line	1/1 disrupted line enhanced tissue overgrowth, 0/1 RNAi line			
RB1		basal del 13q14.2, total del 13q14	del	42.9	*	+	yes	Rbf	1/1 RNAi line, 0/1 amorphic allele line	1/1 line enhanced cell migration	Cosmic, TCGA pan-cancer, Parsons et al, Heterozygous copy loss essential		
ARF6	14	ISAR 64	del	33.3	*	+	no	Arf51F	1/1 RNAi line, 0/2 disrupted lines	1/1 RNAi line enhanced tissue overgrowth	Basal essential		
CREBBP	16	basal del 16p13.3	del	22.9	*	+	yes	nej	1/1 RNAi line	1/1 line enhanced tissue overgrowth	Cosmic, Vogelstein et al		
MAP2K4	17	total del 17p12	del	52.4	*	+	yes	Mkk4	2/4 RNAi lines	1/3 lines enhanced cell migration, 1/2 lines enhanced tissue overgrowth	Cosmic, TCGA pan-cancer, Vogelstein et al, Basal essential		
SERPINB8	18	ISAR 76 basal del 18q23	del	38.1	*	+	yes	Spn55B	1/1 RNAi line	1/1 line enhanced tissue overgrowth			OS 1.48 (0.04)
CCNE1	19	ISAR 78, basal amp 19q12, total amp 19q12	amp	25.7	*	+	yes	CycE	4/6 overexpression lines	1/3 lines enhanced cell migration, 1/2 lines enhanced tissue overgrowth	Cosmic, CiVIC	0	

Table S1. Computational and functional information on each driver gene. * indicates that MYB copy number has a possible negative relationship with expression (Supplemental Table 3) and was tested as both an oncogene and tumor suppressor. † indicates that GRHL2 did not meet criteria for inclusion in the screen but was tested because it shares an ortholog with GRHL1. "t-test Result" refers to a Student's t-test of the effect of copy number on gene expression (see Methods). Genes examined in relevant prior studies (experimental or computational) in breast cancer or other well-known cancer databases are included under "Other Studies": Breast cancer "essential" genes (*41*), COSMIC (*40*), TCGA pan-cancer (*28*), Parsons et al. (*75*), CiVIC (*42*), Myc-synthetic lethal (Myc-SL) (*46*), Aure et al. (*38*), Vogelstein et al.(*56*), TNBC migration driver genes (*47*), TNBC tumor addiction genes (*48*), and driver genes in a mouse model of TNBC (*49*). MutSigCV q-value is shown only for genes with q < 0.1. Progression free interval (PFI) and overall survival (OS) p-values reflect a log-rank test for patients with the aberration vs. without. Only genes with p < 0.1 are shown, all of which the Kaplan-Meier curve plot indicates poorer prognosis with the CNA (Figure 5B-C). See also Supplemental Tables 2, 6, and 7.

Disease Models & Mechanisms • Supplementary information

Table S2. Results of MutSigCV analysis on TCGA breast cancer somatic mutation dataset.

Available for download at

https://journals.biologists.com/dmm/article-lookup/doi/10.1242/dmm.050191#supplementary-data

Table S3. Computational data on all considered GISTIC 2.0 and ISAR genes.

Available for download at

https://journals.biologists.com/dmm/article-lookup/doi/10.1242/dmm.050191#supplementary-data

Table S4. Fly lines and experimental data for tested genes in Group 4G and negative control genes.

Available for download at
https://journals.biologists.com/dmm/article-lookup/doi/10.1242/dmm.050191#supplementary-data

Table S5. Summary of results for all CNA regions considered in this analysis. Regions in gray are not likely to be significant in TNBC, but may be relevant to other breast cancer subtypes; not all of these were studied to completion.

Available for download at
https://journals.biologists.com/dmm/article-lookup/doi/10.1242/dmm.050191#supplementary-data

Table S6. Data from other databases for all considered GISTIC 2.0 and ISAR genes.

Available for download at

https://journals.biologists.com/dmm/article-lookup/doi/10.1242/dmm.050191#supplementary-data

Table S7. Log-rank test hazard ratios (HR) and p-values for driver genes using the TCGA breast cancer dataset. PFI and OS are preferred by the authors of [39] and are the metrics shown in Figure 5. HR are reported as negative for deletions here, and this is corrected in Figure 5.

Available for download at
https://journals.biologists.com/dmm/article-lookup/doi/10.1242/dmm.050191#supplementary-data

Table S8. Drugs used in the drug screen.

Available for download at

https://journals.biologists.com/dmm/article-lookup/doi/10.1242/dmm.050191#supplementary-data

Table S9. Fly stocks and results for Group 1 genes

Results symbols: +: $p<0.05$. ?: $0.05<p<0.4$. -: $p>0.4$ or rescues phenotype. Each symbol represents one experiment.

Available for download at
https://journals.biologists.com/dmm/article-lookup/doi/10.1242/dmm.050191#supplementary-data

Table S10. Fly stocks and results for Group 2 genes

Results symbols: +: $p<0.05$. ?: $0.05<p<0.4$. -: $p>0.4$ or rescues phenotype. Each symbol represents one experiment.

Available for download at
https://journals.biologists.com/dmm/article-lookup/doi/10.1242/dmm.050191#supplementary-data

Disease Models & Mechanisms • Supplementary information

Table S11. Genetic screen results

In the lethality column, only lines that were significant ($p<0.05$) in two independent tests are considered positive and reported in each numerator. In the validation column, 'enhance' refers to affecting an increase in the phenotype over *Myc,p53*^{sh}. Lines were generally only tested for tissue overgrowth (called 'growth' in Supplemental Tables 9, 10) when negative for cell translocation; unless otherwise indicated, a test for tissue overgrowth implies a negative cell translocation test. 'Passenger' refers to genes lacking functional evidence for driver status in this study. ND = not done.

Available for download at
https://journals.biologists.com/dmm/article-lookup/doi/10.1242/dmm.050191#supplementary-data

Table S12. Cell translocation assay results

Stock numbers and statistical results corresponding to Figures 4a and S4a.

Available for download at
https://journals.biologists.com/dmm/article-lookup/doi/10.1242/dmm.050191#supplementary-data

Table S13. Overgrowth assay results

Stock numbers and statistical results corresponding to Figures 4b and S4b.

Available for download at
https://journals.biologists.com/dmm/article-lookup/doi/10.1242/dmm.050191#supplementary-data
